# Comprehensive Analysis of Ubiquitously Expressed Genes in Humans from A Data-driven Perspective

**DOI:** 10.1016/j.gpb.2021.08.017

**Published:** 2022-05-13

**Authors:** Jianlei Gu, Jiawei Dai, Hui Lu, Hongyu Zhao

**Affiliations:** 1 SJTU-Yale Joint Center for Biostatistics and Data Science, Department of Bioinformatics and Biostatistics, School of Life Sciences and Biotechnology, Shanghai Jiao Tong University, Shanghai 200240, China; 2 Center for Biomedical Informatics, Shanghai Engineering Research Center for Big Data in Pediatric Precision Medicine, Shanghai Children’s Hospital, Shanghai 200040, China; 3 Department of Biostatistics, Yale University, New Haven, CT 06511, USA

**Keywords:** Ubiquitous expression, Housekeeping gene, Disallowed gene, Expression specificity, Expression variability

## Abstract

Comprehensive characterization of spatial and temporal gene expression patterns in humans is critical for uncovering the regulatory codes of the human genome and understanding the molecular mechanisms of human diseases. Ubiquitously expressed genes (UEGs) refer to the genes expressed across a majority of, if not all, phenotypic and physiological conditions of an organism. It is known that many human genes are broadly expressed across tissues. However, most previous UEG studies have only focused on providing a list of UEGs without capturing their global expression patterns, thus limiting the potential use of UEG information. In this study, we proposed a novel data-driven framework to leverage the extensive collection of ∼ 40,000 human transcriptomes to derive a list of UEGs and their corresponding global expression patterns, which offers a valuable resource to further characterize human transcriptome. Our results suggest that about half (12,234; 49.01%) of the human genes are expressed in at least 80% of human transcriptomes, and the median size of the human transcriptome is 16,342 genes (65.44%). Through gene clustering, we identified a set of UEGs, named LoVarUEGs, which have stable expression across human transcriptomes and can be used as internal reference genes for expression measurement. To further demonstrate the usefulness of this resource, we evaluated the global expression patterns for 16 previously predicted **disallowed genes** in islet beta cells and found that seven of these genes showed relatively more varied expression patterns, suggesting that the repression of these genes may not be unique to islet beta cells.

## Introduction

In multicellular organisms, different tissues or cells contain mostly the same genome. However, each tissue or cell type only expresses a subset of its genes and has its own unique transcriptome. The variations among transcriptomes underlie the wide range of phenotypic and physiologic differences across tissues or cells [1]. It is generally believed that the genes within a transcriptome could be broadly divided into two groups: the ubiquitously expressed genes (UEGs), traditionally called housekeeping (HK) genes [2], and the specifically expressed genes (SEGs) [3]. UEGs are expressed in almost all living cells of an organism and play an essential role in maintaining cellular processes and cell survival. On the other hand, SEGs are strictly expressed in a limited number of tissue or cell types and usually have specific biological functions. They are generally believed to be more likely associated with human diseases and/or druggable targets [4]. The more recent view of UEGs or HK genes has emphasized that these genes should be insensitive to cell type heterogeneity and have stable expression across tissues [5], [6]. In this study, we used the term UEGs rather than HK genes to describe those widely expressed genes with some having variations across conditions, and systematically characterized the global expression patterns of UEGs in the human genome.

Much work has been conducted to characterize the UEGs in the human genome [5], [7], [8], [9], [10]. However, the reproducibility of the UEG lists from early studies was low due to the limitations of microarray techniques [6]. As far as we know, it was not until 2008 that Jiang et al. [9] first reported that there might be a large number of human genes (about 40% of human genes) broadly expressed across tissues through the analysis of an expressed sequence tag (EST) data collection. With the development of the RNA-seq technology, this observation was substantiated by RNA-seq studies [7], [8], with approximately 8000 to 10,000 genes broadly expressed across tissues. However, there are several limitations in the published UEG studies. First, there are over 200 tissue/cell types in the human body, and there can be substantial variations in transcriptomes across biological conditions and individuals [6]. The published UEG studies are often limited in the number of tissue and cell types covered. Second, published UEG studies often use a single tissue-specificity measure of expression to identify UEGs and do not fully capture gene expression patterns, thus limiting the potential use of UEG information. Although some UEG studies have considered expression variability, it has only been used as a hard filtering criterion [5]. For bulk RNA-seq data, the observed expression level for each gene is the aggregated expression value of a large number (maybe heterogeneous) of cells. Thus, traditional bulk RNA-seq data offer a bird’s-eye view of the expression patterns at the cell population level.

**Figure 1:**
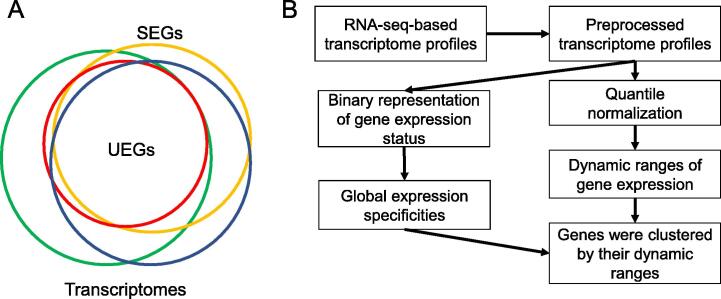
**The flow diagram for systematic characterization ofUEGsin the human genome** **A.** Definition of global expression specificity. Different colored circles represent the transcriptomes derived from different tissues or cell types. The overlapping area represents the core component of human transcriptomes, *i.e.*, the UEGs. **B.** After preprocessing, we performed a sample-wise quantile normalization that allowed us to obtain a robust global distribution of expression levels for each gene. Then, we clustered genes by their dynamic ranges of global distribution. Finally, the global expression specificity metrics were mapped to the genes and gene clusters. SEG, specifically expressed gene; UEG, ubiquitously expressed gene.

Inspired by the concept of pan-genome and core-genome in bacterial research [11], [12], we hereby proposed a novel analysis framework to systematically characterize human UEGs, which represents the core component of human transcriptomes. Through simultaneous consideration of a large collection of diverse transcriptomes, our framework bypassed the subjective tissue/cell type stratification process to directly assess the global expression specificity and the expression pattern for each gene (Figure 1). By analyzing ∼ 40,000 divergent human transcriptomes, we observed that 12,234 human genes (49.01%) are ubiquitously expressed in at least 80% of human transcriptomes, and the median size of the human transcriptome is 16,342 genes (65.44%). Coupled with global expression patterns of these genes, we identified a set of UEGs, named LoVarUEGs, which have stable expression across biological conditions and can be used as internal reference genes for expression measurement. Collectively, as a separate validation, we observed similar results in another RNA-seq data repository, DEE2 [13], supporting the generalizability of our findings. To demonstrate the usefulness of our UEG resource, we evaluated the global expression patterns of 16 previously predicted disallowed genes in pancreatic islet beta cells, and found that seven of these putative disallowed genes had more varied expression patterns than classical disallowed genes, suggesting that the repression of these genes may not be unique to islet beta cells, at least in term of expression level. In summary, our study provides a useful framework and resource for further functional genomics studies of human genes.

## Results

### Highly phenotypic heterogeneity of analyzed transcriptomes

**Figure 2:**
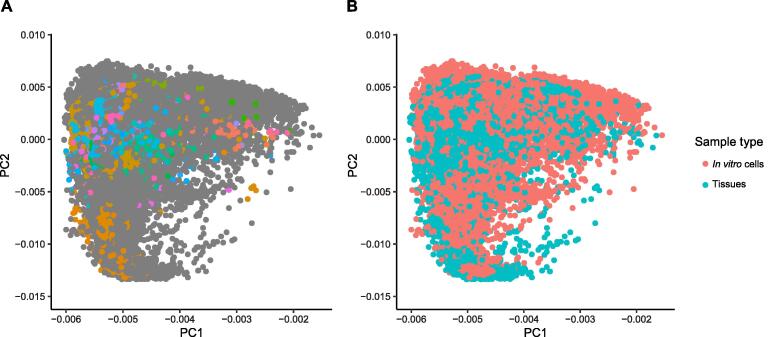
**The phenotypic compositions of analyzed transcriptomes** The onlinePCA (https://cran.r-project.org/package=onlinePCA) was performed to the quantile-normalized expression matrix to visualize the phenotypic compositions and relatedness among transcriptomes. Each dot represents one transcriptome projected on the principal plane formed by the first and second principal axes. **A.** Diversity of phenotypes in transcriptome profiles. The colored dots represent the 6501 (16.31%) manually curated reference transcriptomes belonging to 101 tissue groups. Gray dots represent those unclassified transcriptomes that exhibit a broad spectrum of heterogeneity. **B.** Transcriptome profiles generated from *in vitro* and *in vivo* samples. The cyan dots represent the transcriptomes from tissue samples, and the red dots represent the transcriptomes from *in vitro* cells. PC, principal component.

In this study, we primarily used the recount2 repository [14], [15], which comprises ∼ 50,000 RNA-seq based human transcriptome profiles. After preprocessing (described in Materials and methods), 39,863 (80.3%) transcriptome profiles were retained for further analyses. We annotated the tissue types of these transcriptome profiles with an automated semantic annotation database [16]. These transcriptomes covered more than 30 organ systems, with musculoskeletal system (10.09%) being the most common tissue, followed by hemolymphoid system (8.86%), nervous system (7.61%), and digestive system (2.74%) (Table S1). To improve sample coverage across more conditions, we also included the transcriptomes from *in vitro* cells [including cell lines, primary cells, *in vitro* differentiated cells, stem cells, and induced pluripotent stem (iPS) cells], being about 56.7% of the total samples (Table S2). For reference, we further manually annotated 6501 (16.31% of total) transcriptomes that represent 101 major tissue types (Table S3). To check the relatedness among these transcriptomes, we used onlinePCA (https://cran.r-project.org/package=onlinePCA) to visualize the first two principal components (PCs) of all 39,863 transcriptome profiles (Figure 2). We can see that these divergent transcriptomes collected from various experiments were reasonably clustered, and those unclassified transcriptomes exhibited a broad transcriptomic heterogeneity. In addition, we found that 17,503 (43.91%) transcriptomes showed relatively high relatedness (Figure S1), suggesting that these transcriptomes may be overrepresented in the recount2 dataset. We then conducted a sensitivity analysis to evaluate the impact of these overrepresented samples (described in File S1) and observed that these overrepresented transcriptomes had limited effects on our overall results and conclusions (Figures S2–S4). To evaluate the generalizability of our results, we applied our analysis framework to a more recent transcriptome dataset, DEE2 [13], which is a public repository of uniformly processed RNA-seq profiles. The differences between DEE2 and recount2 datasets are that (1) they used different pipelines to generate transcriptome profiles; and (2) they only shared about 15% of the samples and had different relatedness patterns (Figure 2, Figures S1 and S5).

### The majority of human genes are either ubiquitously or specifically expressed

**Figure 3:**
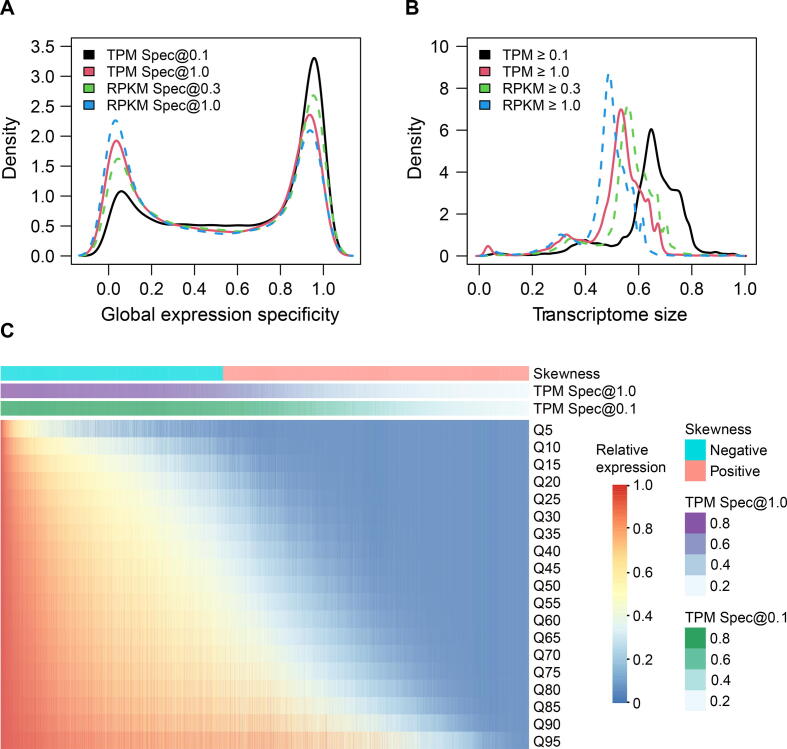
**The global expression specificity and global dynamic ranges of expression values** **A.** Density plot showing the global expression specificity obtained at different detection thresholds (TPM ≥ 0.1, TPM ≥ 1.0, RPKM ≥ 0.3, and RPKM ≥ 1.0). TPM Spec@0.1, TPM Spec@1.0, RPKM Spec@0.3, and RPKM Spec@1.0 donate the global expression specificity determined by the thresholds of TPM ≥ 0.1, TPM ≥ 1.0, RPKM ≥ 0.3, and RPKM ≥ 1.0, respectively. **B.** Density plot showing the distribution of human transcriptome size detected at different thresholds (TPM ≥ 0.1, TPM ≥ 1.0, RPKM ≥ 0.3, and RPKM ≥ 1.0). **C.** Quantile-normalized transcriptome profiles are summarized into dynamic ranges [the lowest 5% (Q5) to highest 95% (Q95) relative expression level] and used to generate a heatmap showing the global expression pattern for each gene. TPM, transcripts per million; RPKM, reads per kilobase of transcript per million reads mapped.

We proposed to use the proportion of samples in which a gene was expressed across all the transcriptomes to quantify its expression specificity. We referred to this proportion as global expression specificity ϕ, where ϕ = 1 denotes a UEG and ϕ close to 0 denotes a highly expressed SEG. As shown in Figure 3A, the distribution of ϕ had a clear bimodal distribution, *i.e.*, most genes were either ubiquitously or specifically expressed, which is consistent with previous observations [17]. In order to determine the optimal expression detection threshold, we made a comparison between four commonly used detection thresholds and found that the threshold of transcripts per million (TPM) ≥ 0.1, which was used in the GTEx project, was a robust and sensitive detection threshold for those lowly expressed genes (Figure S6; Table S4). Applying this threshold, 12,267 (49.14%) genes had their ϕ ≥ 0.8 and 7439 (29.80%) genes had ϕ ≤ 0.4 (Table 1). To compare ϕ with traditional tissue-based expression specificity using manually curated samples with tissue information, we calculated tissue-based specificity and compared these two metrics across genes. As shown in Figure S7, these two metrics were highly correlated with a Pearson correlation coefficient (PCC) of 0.960. Only 2279 genes (9.1%) had a difference ≥ 0.2 (20% of total range) between these two specificity metrics, where genes with relatively high or low global expression specificity had a higher agreement (Figure S8). Moreover, with this detection threshold, we found that 80% of the human transcriptomes had 11,166 (44.71%) to 19,033 (76.21%) expressed genes, and the median number of expressed genes was 16,342 (65.44%) (Figure 3B). This number was close to what was reported in a smaller-scale study [8]. This suggests that the average difference in gene content between human transcriptomes is only 16.43%.

**Table 1:** **The number of genes in each specificity interval**

	**Global expression specificity**					
	**0.8**–**1.0**	**0.6**–**0.8**	**0.4**–**0.6**	**0.2**–**0.4**	**0**–**0.2**	**Total**
All genes	12,267 (49.14%)	2727 (10.92%)	2530 (10.13%)	2641 (10.58%)	4798 (19.22%)	24,963
Skewness **≤** 0	10,421 (99.20%)	84 (0.80%)	0 (0%)	0 (0%)	0 (0%)	10,505 (42.08%)
Q10 ≥ 0.1	9692 (100%)	0 (0%)	0 (0%)	0 (0%)	0 (0%)	9692 (38.83%)
Q20 ≥ 0.1	12,002 (99.98%)	3 (0.02%)	0 (0%)	0 (0%)	0 (0%)	12,005 (48.09%)
2011 UEG ARRAY [10]	2038 (99.66%)	4 (0.20%)	2 (0.10%)	1 (0.05%)	0 (0%)	2045 (8.19%)
2009 UEG SEQ [8]	7703 (98.88%)	59 (0.76%)	13 (0.17%)	13 (0.17%)	2 (0.03%)	7790 (31.21%)
2014 UEG SEQ [7]	8696 (97.60%)	176 (1.98%)	37 (0.42%)	0 (0%)	1 (0.01%)	8910 (35.69%)
2013 HK SEQ [5]	3786 (99.82%)	5 (0.13%)	2 (0.05%)	0 (0%)	0 (0%)	3793 (15.19%)
BodyMap SEGs [3]	735 (20.75%)	573 (16.17%)	708 (19.98%)	808 (22.81%)	719 (20.29%)	3543 (14.19%)
GTEx SEGs [3]	1128 (27.96%)	661 (16.39%)	748 (18.54%)	806 (19.98%)	691 (17.13%)	4034 (16.16%)
Essential genes [26]	5356 (77.00%)	548 (7.88%)	430 (6.18%)	345 (4.96%)	277 (3.98%)	6956 (27.87%)
Trait genes [27]	2246 (72.33%)	372 (11.98%)	251 (8.08%)	172 (5.54%)	64 (2.06%)	3105 (12.44%)
Genetic disease genes [28]	9759 (61.42%)	1862 (11.72%)	1644 (10.35%)	1443 (9.08%)	1180 (7.43%)	15,888 (63.65%)
DRUGABLE genes [33]	1764 (40.84%)	711 (16.46%)	738 (17.09%)	643 (14.89%)	463 (10.72%)	4319 (17.30%)
UEGs@1.0 category	9687 (100%)	0 (0%)	0 (0%)	0 (0%)	0 (0%)	9687 (38.81%)
UEGs@0.1 category	2272 (89.20%)	275 (10.80%)	0 (0%)	0 (0%)	0 (0%)	2547 (10.20%)
MEG category	307 (9.75%)	2094 (66.48%)	749 (23.78%)	0 (0%)	0 (0%)	3150 (12.62%)
SEGs@1.0 category	1 (0.03%)	358 (11.71%)	1771 (57.91%)	928 (30.35%)	0 (0%)	3058 (12.25%)
SEGs@0.1 category	0 (0%)	0 (0%)	10 (0.15%)	1713 (26.27%)	4798 (73.58%)	6521 (26.12%)

*Note*: “All genes” indicates total genes analyzed in this study; “UEG SEQ” indicates UEGs derived from an RNA-seq-based study; “HK SEQ” indicates HK genes derived from an RNA-seq-based study which takes into account the variability of gene expression; “UEG ARRAY” indicates UEGs derived from a microarray-based study. Q10, 10% percentile; Q20, 20% percentile; UEG, ubiquitously expressed gene; SEG, specifically expressed gene; MEG, moderately specific gene; HK, housekeeping.

### Distribution skewness of relative expression values is strongly associated with global expression specificity

Although it is useful to classify genes into UEG and SEG groups by their expression specificity, such classification cannot capture global expression patterns. Through joint analyses of diverse transcriptomes, we can characterize the dynamic ranges of relative expression values for each gene. To reduce batch effects in defining global expression patterns, we used a sample-wise quantile transformation [18] to TPM- or reads per kilobase of transcript per million reads mapped (RPKM)-normalized transcriptome profiles. After transformation, expression values were replaced by their percentile ranks for each profile. Figure 3C displays the dynamic ranges of gene expression values [the lowest 5% (Q5) to the highest 95% (Q95) relative expression values for each gene]. With an empirical threshold of 10% percentile (Q10) **≥** 0.1 (Figure S9), the expression levels of 9692 (38.83%) genes were above Q10 in at least 90% of all transcriptomes. With a more relaxed threshold of 20% percentile (Q20) ≥ 0.1, this number increased to 12,005 (48.09%), *i.e.*, these genes’ expression levels were above Q20 in at least 80% of the samples (Table 1). These observations were close to the inference of UEGs through ϕ. Table S5 lists all the genes with their global expression specificity and dynamic ranges.

**Figure 4:**
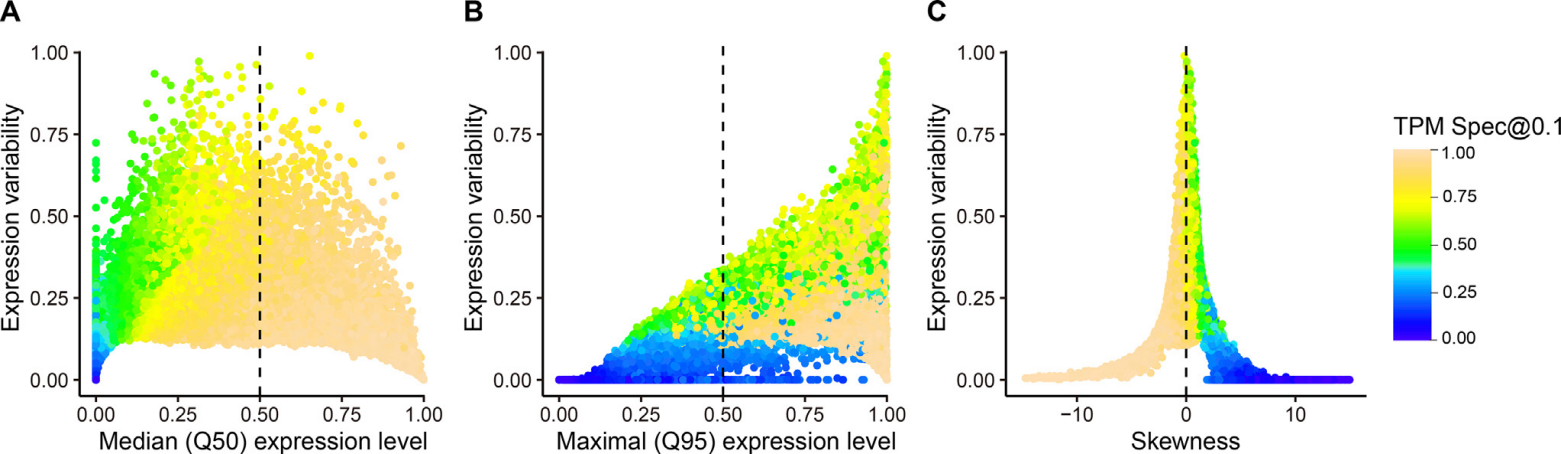
**The relationship between expression specificity, expression level, and expression variability** **A.** Scatter plot showing the relationship between median (Q50) expression level, expression specificity, and expression variability. **B.** Scatter plot showing the relationship between maximal (Q95) expression level, expression specificity, and expression variability. **C.** Scatter plot showing the relationship between distribution skewness, expression specificity, and expression variability.

We then examined the relationships between global expression specificity and the distribution attributes of relative expression values, including mean, median, interquartile range (IQR; variability), and skewness. As expected, the genes with larger ϕ tended to have higher median (Q50) and maximal (Q95) expression levels (Figure 4A and B), which is consistent with previous observations [17], [19], [20]. One interesting finding is that the global expression specificity is strongly associated with distribution skewness of relative expression values as observed in previous tissue specificity of gene expression (Spearman correlation coefficient is −0.97) [17], [21]. The UEGs were enriched with genes showing negative skewness, whereas the SEGs were enriched with genes having positive skewness (Figures 3C and 4C; Table 1). About 90% of previously reported UEGs had a negatively skewed distribution, and ∼ 80% of previously reported SEGs had a positively skewed distribution.

### Global expression specificity categories and functional implications

**Figure 5:**
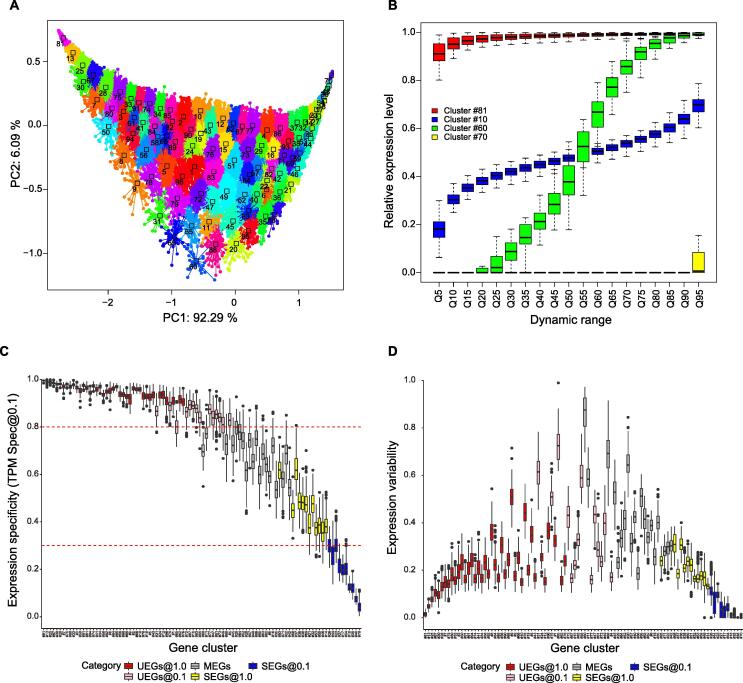
**Global expression specificity categories** **A.** PCA plot visualizing the global expression patterns and clustering results. Each dot represents a gene. Different colors represent 96 gene clusters. The genes within the same cluster show similar expression levels, expression specificity, and expression variability. **B.** Boxplot showing the global expression patterns of some gene clusters. **C.** Boxplot showing the distribution of expression specificity of 96 clusters. The clusters (boxes) are ranked according to their median skewness. The red dashed lines represent the global expression specificity (ϕ) of 0.8 (upper) and 0.3 (lower), respectively, using the detection threshold of TPM ≥ 0.1. **D.** Boxplot showing the distribution of expression variability among these clusters. The clusters (boxes) are ranked according to their median skewness. PCA, principal component analysis.

To group genes according to their global expression patterns, we performed clustering on the dynamic range matrix through percentile clustering [22], that is, to cluster genes according to their summarized distribution shapes of expression values. After clustering, the genes with similar expression levels, expression variability, and expression specificity were grouped into the same cluster (Figure 5). Figure 5A shows the principal component analysis (PCA) plot of the dynamic range matrix with 96 gene clusters inferred by the affinity propagation clustering method [23]. Figure 5B shows the dynamic range for some gene clusters, *e.g.*, cluster #81 with most ubiquitously and highly expressed genes, cluster #10 with ubiquitously but lowly expressed genes, cluster #60 with the most varied expression pattern, and cluster #70 with the most restricted expression pattern. We then mapped the global expression specificity to these gene clusters, and with such information, we can broadly classify these gene clusters into five specificity categories (Figure 5C): (1) UEGs@1.0, a UEG category detected by the threshold of TPM ≥ 1.0 (with median ϕ of clusters **≥** 0.8). This category included 9687 (38.81%) genes in 40 clusters that had a ubiquitous expression pattern. The genes in this category are more likely involved in essential cellular processes, such as transcription (11.95%), apoptotic process (3.45%), oxidation–reduction process (3.28%), protein transport (3.16%), and cell division (2.95%). (2) UEGs@0.1, a UEG category only inferred by a more sensitive detection threshold of TPM ≥ 0.1 (with median ϕ of clusters ≥ 0.8). This category was composed of 14 clusters involving 2547 (10.20%) genes. Some clusters in this category showed low expression levels, *e.g.*, cluster #77 and cluster #4, and they might be easily overlooked by stringent detection thresholds (Figure S6; Table S4) or experiments with insufficient detection sensitivity. A total of 352 (78.40%) genes of these two gene clusters were above Q10 in at least 80% of all transcriptomes studied, whereas only 4.45%−6.46% of them were classified as UEGs in previous UEG studies [7], [8]. On the other hand, some clusters in this category showed relatively higher expression variability, *e.g.*, clusters #31, #65, and #55. Although these genes were widely detectable, their percentile ranks within each transcriptome varied significantly across biological conditions. This means that these gene clusters with higher expression variability are more likely to have a leaky expression [5], [24] and are more sensitive to biological conditions. (3) Moderately specific gene (MSG) category. This category included 3150 (12.62%) genes in 20 gene clusters. The genes in this category are mainly involved in the regulation of biological processes, including signal transduction (7.59%), cell adhesion (4.66%), and inflammatory response (3.89%). (4) SEGs@1.0, an SEG category detected by the threshold of TPM ≥ 1.0 (with median ϕ of gene clusters **≤** 0.3). This category included 3058 (12.25%) genes in 12 clusters. (5) SEGs@0.1, an SEG category only detected by the threshold of TPM ≥ 0.1 (with median ϕ of clusters **≤** 0.3). This category included 6521 (26.12%) genes in 10 clusters.

The SEG categories SEGs@1.0 and SEGs@0.1 refer to those genes that are specifically expressed in a limited set of biological conditions and have specialized functions. The genes in these two specific categories are likely involved in various specific biological processes, such as G-protein coupled receptor signaling pathway (6.58%), sensory perception of smell (4.13%), multicellular organism development (1.88%), and proteolysis (1.72%). All functional enrichment results are listed in Table S6.

### A large fraction of UEGs involves human diseases

Since UEGs play an essential role in maintaining cellular processes and cell survival, they have been considered unlikely to be a disease gene, especially for genetic diseases [25]. We observed that approximately 80% of the essential genes [26] exhibited a ubiquitous expression pattern (ϕ ≥ 0.8, Table 1). However, we compared ϕ with the genes associated with physiological traits [27] and genetic diseases [28], and observed that about 70% of physiological trait-related or disease-related genes exhibited a ubiquitous expression pattern (ϕ ≥ 0.8, Table 1). For example, loss-of-function mutations in the *ACTB* gene, a most abundant cytoskeletal HK gene, cause development disorder and intellectual disability [29]; expanded trinucleotide repeats in the *TBP* gene, encoding an important general transcription initiation factor, cause a Huntington disease-like phenotype [30], [31], [32]. Our results indicate that even the most UEGs cannot be simply ignored during the prioritization of causal genes/variants. On the other hand, genes with restricted expression patterns are believed to be good drug targets due to improved efficacy and safety [4]. This is supported by our observation that 59.16 % of the reported druggable genes [33] show significantly varied (ϕ < 0.8) expression levels between biological conditions (Table 1).

### Evaluation of the global expression patterns of disallowed genes

**Figure 6:**
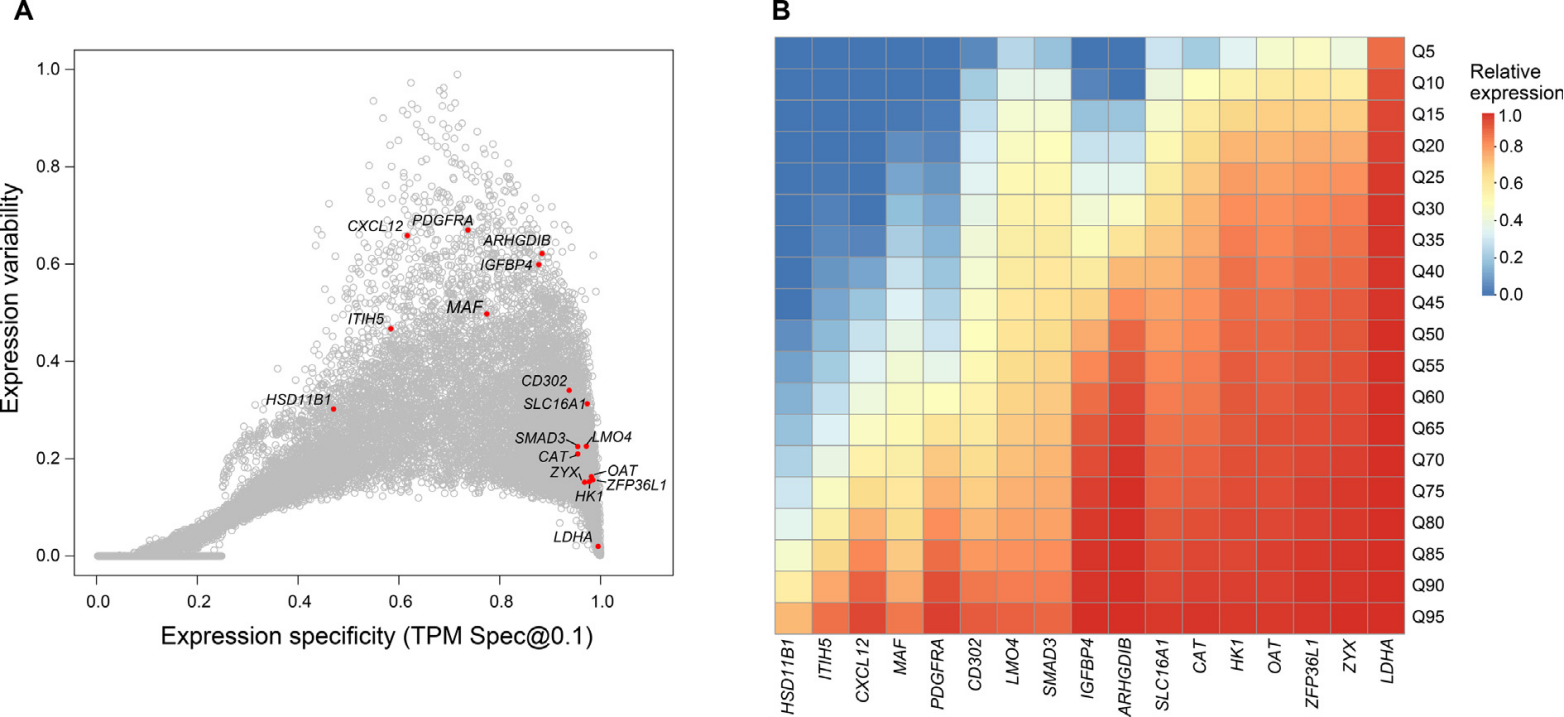
**Evaluation ofthe global expression patternsofputative disallowed genes in isletbeta cells** **A.** The global expression specificity and variability among reported disallowed genes. **B.** The global dynamic ranges of expression levels among reported disallowed genes.

An interesting example of UEGs associated with vital physiological phenotypes is the important metabolic enzyme gene *LDHA* and the *SLC16A1* gene encoding a transporter MCT-1, which belong to a class of so-called disallowed genes which were first described in the beta cells of pancreas islets [34]. In contrast to SEGs, disallowed genes refer to those UEGs that are specifically repressed only in a few cell types and with likely functional consequences [34], [35]. For example, the inactivation of *LDHA* and *SLC16A1* plays a critical role in the maturation of beta cells and the secretion of insulin. The aberrant activation of *LDHA* or *SLC16A1* has been observed to cause diabetes-like phenotype or exercise-induced hyperinsulinism (EIHI). Following the success of *LDHA* and *SLC16A1*, a number of putative disallowed genes have been reported [35], [36], [37]. Although the repression stability of some putative disallowed genes has been extensively validated [37], they have not been validated from the perspective of UEGs, *i.e.*, the uniqueness of the repression. We think this is partly due to the lack of a reliable UEG list and corresponding global expression patterns. The identification and validation of disallowed genes can be viewed as a special application of outlier analysis [38], [39]. Our study provides a resource to evaluate the uniqueness of repression for putative disallowed genes. As shown in Figure 6, the classic disallowed genes *LDHA* and *SLC16A1* exhibited strong constitutive expression patterns across a large collection of transcriptomes. Even the *HK1* gene, which is specifically repressed in beta cells and liver cells and does not fulfill the strictest definition of disallowance [35], also showed a strong constitutive expression pattern. However, some putative disallowed genes exhibited a significantly restricted expression pattern, such as *ITIH5*, *CXCL2*, and *HSD11B1*. For example, the *HSD11B1* gene showed a relatively restricted expression pattern in the adrenal gland (expressed in 22.58% samples) and bone marrow (expressed in 33.70% samples). Besides, although the genes *IGFBP4*, *MAF*, *PDGFRA*, and *ARHGDIB* had a ubiquitous expression pattern, their relative expression levels showed significant differences across biological conditions. In addition, these observations were replicated in the DEE2 dataset (Figure S10). These results suggest that, unlike the classic disallowed genes, the repression of these genes may not be unique to the islet beta cells, and the function of their repression may need more detailed investigation.

## Discussion

The goal of our work is to identify and characterize the core genes in the human transcriptome, a long-standing problem in functional genomics. Earlier studies of UEGs relied heavily on the tissue-stratification strategy and were limited in sample size, which resulted in low consistency across studies and failure of capturing global expression patterns, and thus limited the potential use of UEG information. As by definition, UEGs should be present across a majority of, if not all, phenotypic and physiological conditions of an organism, and the laborious and error-prone annotation/curation process may be overcome by the use of a diverse and extensive collection of transcriptomes. In this study, we proposed a global expression specificity metric that used the proportion of samples in which a gene was expressed across a large collection of diverse transcriptomes to represent its global expression specificity. Comparisons with results based on tissue-specific expression patterns showed that the global expression specificity was highly concordant with tissue-specific results (Figures S7 and S8) and was also robust to uneven distribution of samples across tissue types in the repositories (Figure S2).

Leveraging diverse transcriptome profiles, we can establish the global distribution of relative expression values (Figure 3C) for each human gene, and this information can be used to further validate and characterize human UEGs. We examined the relationships between global expression specificity and global distribution attributes of relative expression values. We observed that the UEGs with higher expression levels usually had relatively lower variability in percentile rank within the transcriptome. However, a number of studies found that even for those most commonly used internal reference genes, there was often considerable expression variability across biological conditions [40], [41], [42], [43]. About 55.98% of UEGs, especially those highly expressed UEGs, exhibited a narrow distribution of relative expression values, which are correlated with low expression variability (0–0.2) (Table S7). This suggests that most UEGs maintain relatively constant percentile rank within transcriptomes across divergent biological conditions and can serve as good candidates for internal references in most cases. On the other hand, lowly expressed UEGs exhibited relatively higher variability, partly because the percentile ranks of lowly expressed genes were more likely affected by other genes and the size of transcriptomes. In fact, the variability of the observed expression values of a gene was positively correlated with its expression magnitudes (Figure S11).

**Figure 7:**
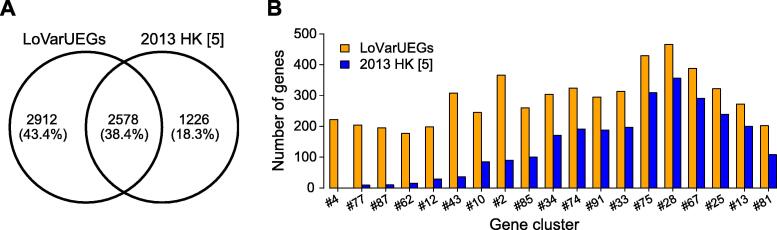
**Comparison ofLoVarUEGs with the previously reported HK genes with stable expression** **A.** Venn diagram showing the overlap between LoVarUEGs and the previously reported HK gene with stable expression (2013 HK [5]). **B.** Number of genes in the 19 UEG clusters in LoVarUEGs as well as in 2013 HK [5]. X-axis indicates the gene clusters ordered by median expression levels, from left (lowly expressed) to the right (highly expressed). The details of the 19 UEG clusters in the LoVarUEGs set are listed in Table S4. LoVarUEGs indicates a set of UEGs which have stable expression across human transcriptomes analyzed in this study.

To better characterize the overall expression patterns for all human genes, we clustered them into clusters, where genes in the same cluster had similar expression levels, expression variability, and expression specificity (Figure 5). With the help of these gene clusters, we identified 19 UEG clusters containing 5671 genes (*i.e.*, LoVarUEGs) with low variability in expression levels. We then checked their dynamic ranges of raw TPM values in both the recount2 and DEE2 datasets and confirmed their ubiquitous and stable expression patterns (Figure S6; Tables S8 and S9). After removing outliers (about 3.19%), 5490 genes had relatively stable TPM values across human transcriptomes and can be used as internal reference genes for expression measurement (Table S4). Compared with previously reported HK genes with stable expression [5], these genes had comparable stability of expression in both the recount2 and DEE2 datasets (Figures S11 and S12). Nevertheless, the LoVarUEGs showed significantly better coverage for lowly expressed genes (Figure 7). As an advantage over previous studies, our study stratified stably expressed UEGs by their overall expression patterns so that they can be easily selected and used as internal references for various downstream applications [44]. For example, in this work, we used the lowly expressed UEG clusters to evaluate and determine the optimal expression detection threshold. Interestingly, when we mapped the expression stability of the single cells [45] onto our gene clusters, we observed that the sparsity (fraction of zeros) of single-cell profiles highly correlated with the global expression specificity and the expression magnitudes at bulk level (Figure S13). The stably expressed UEG clusters with higher expression levels showed lower single-cell sparsity and *vice versa*. This implies that these stably expressed UEG clusters might be a good model to study potential connections in gene expression between bulk and single-cell levels, which may be useful for cell-type deconvolution [46] and adjusting potential dropout bias [47]. Moreover, these gene clusters provide local context information for transcriptome profiles that can further improve the outlier analysis approaches [18], [38].

As a validation of our results, we applied our analysis framework to a more recent RNA-seq dataset, DEE2 [13]. As shown in Figure S14, the global expression specificity metric was highly reproducible between these two datasets (PCC = 0.937). Only 5.7% of genes had global expression specificity differences greater than 0.2, and some differences may be caused by different profiling pipelines or gene annotations. In addition, the global distribution attributes for each gene were also highly consistent (Figures S15 and S16). Finally, a total of 86.2% of UEGs generated from these two repositories overlapped (Figure S17). Comparisons with previous UEG and SEG studies (Table 1) showed that (1) early microarray-based UEG studies significantly underestimated the number of human UEGs; (2) over 95% of previously reported UEGs were validated in our study (ϕ ≥ 0.8); (3) 2804 novel UEGs were identified in this stuty, 73.57% of which were also found in the separate dataset DEE2 (Figure S17); (4) there was a significant overlap between UEGs and SEGs. About 37%−43% of previously reported SEGs had a strict specific expression pattern (ϕ ≤ 0.4), but about 21%−28% of these reported SEGs exhibited a ubiquitous expression pattern (ϕ ≥ 0.8). It implies that some genes may be both ubiquitously and tissue-enriched expressed. For example, the lipid transport gene *APOE*, which is a major risk gene for Alzheimer’s disease [48], showed high expression variability (0.74) while being widely expressed (ϕ = 0.87), and this gene has been labeled as both a UEG or SEG by several studies [3], [7], [8]. The *ALAS1* gene, which is a widely used internal reference gene, was classified as a tissue-specific gene in two recent SEG studies [3], [4]. In addition, we found that even using the same analysis method [3], there was only 38.6% overlap of the identified SEGs between GTEx and BodyMap datasets (Figure S18). Collectively, these observations suggest that a comprehensive study for human SEGs is still required.

Generally, UEGs should be expressed in all living cells of an organism. However, a specific subset of UEGs, called disallowed genes [34], are selectively repressed in some specific cell types and with likely functional consequences. *LDHA* and *SLC16A1* are the most well-studied disallowed genes in the pancreatic islet beta cells [49]. The repression of *LDHA* is thought to be crucial for the maturation of beta cells and the secretion of insulin. The beta cells in diabetes models show loss of repression and up-regulated expression of *LDHA*. The repression of *SLC16A1* prevents the inappropriate stimulation of insulin release during physical exercise, and correspondingly, aberrant activating *SLC16A1* results in EIHI [50], [51]. The identification of disallowed genes in beta cells has raised the interesting question of whether there are other disallowed genes in beta cells or other cell types [49], [52]. Our study provides a comprehensive UEG resource that could be used to evaluate the uniqueness of repression for the identification and validation of disallowed genes. To demonstrate, we evaluated 16 putative disallowed genes in beta cells [35], [37] and found that seven of them (Figure 6, Figure S10), including *HSD11B1*, *ITIH5*, *CXCL12*, *IGFBP4*, *PDGFRA*, *MAF*, and *ARHGDIB*, exhibited relatively more varied expression patterns. Although our observation is limited to expression level through the UEG perspective, it may offer a new angle for these genes in beta cells. Moreover, a recent single-cell study revealed that even for the most common UEGs, such as *GAPDH* and *ACTB*, they showed a clear repression pattern in some cells [45]. This implies that repression of gene expression at the single-cell level is likely a common regulatory mechanism, and more disallowed genes might exist in specific cell types.

In summary, we have presented a novel data-driven framework that uses a large collection of transcriptomes to systematically characterize UEGs. As a major improvement over previous studies, we provide the global expression patterns for human genes that can be used to further validate and characterize UEGs. We have also explored some potential functional implications of UEGs in biomedical research and offered an interesting example to demonstrate the usefulness of this resource in the evaluation of disallowed genes.

## Materials and methods

### Preprocessing and phenotypic annotation for transcriptome profiles

We downloaded 49,649 human transcriptome profiles at the gene level from the recount2 repository [14], [15]. As the original profiles used Ensembl gene ID, we converted Ensembl ID into Entrez ID by the Ensembl BioMart tool (Table S10). If multiple transcripts matched a single Entrez ID, we used the maximum value of these transcripts to represent the expression level of this gene. The gene-level expression matrix was further normalized by TPM and RPKM [53]. In the following analyses, we found that the TPM threshold had better detection sensitivity, so that the main results were analyzed by TPM-normalized data and the RPKM-based results were only used for comparison. Because of potential quality issues for transcriptome profiles derived from disparate experiments, we filtered low-quality transcriptome profiles by the following criterion. If the expression measurement of any of three lowly expressed internal reference genes (*GUSB*, *HPRT1*, and *HMBS*) in a transcriptome is zero, this transcriptome was considered to be low quality and excluded from further analyses (Figure S19). After removal, a total of 39,863 (80.3%) transcriptome profiles remained for further analyses. The DEE2 dataset [13] provided the quality control information for each profile, with a total of 61,020 high-quality DEE2 transcriptome profiles, which were labeled as ‘PASS’ and were used for further analysis. We used the same preprocessing method to convert the original profiles into TPM- and RPKM-normalized matrices.

To check the phenotypic composition of these transcriptomes, each transcriptome was labeled with a series of biomedical ontology terms by an automatic semantic annotation database MetaSRA [16], and the sample type was also predicted by MetaSRA. Moreover, we manually annotated 6501 (16.31%) reference transcriptomes with 101 tissue types and visualized these reference transcriptomes and unclassified transcriptomes by the onlinePCA package in R (https://cran.r-project.org/package=onlinePCA). The manually annotated information of these transcriptomes is listed in Table S3. The overrepresented samples were identified by the PCA ordination density plot with manually determined cutoffs (Figure S1).

### Traditional tissue-based expression specificity and global expression specificity

Traditionally, UEG studies use a tissue stratification strategy to determine the tissue specificity of gene expression in order to identify UEGs. This strategy is useful with a limited number of tissue groups and sample size. In this study, we used the proportion of tissues expressing each gene to represent the traditional tissue specificity of expression [17]. The manually curated subset was used to calculate tissue specificity for a gene:

(1) $$\mathrm{Ti}ssue\;\mathrm{specificity}=\frac{\mathrm{Number}\;\mathrm{of}\;\mathrm{tissues}\;\mathrm{expressing}\;\mathrm{this}\;\mathrm{gene}}{\mathrm{Number}\;\mathrm{of}\;\mathrm{total}\;\mathrm{tissues}}$$

We used TPM ≥ 0.1 as the expression detection threshold for each sample. Since there are multiple samples belonging to each tissue group, we used 80% as a cutoff to determine whether this tissue expressed this gene.

However, when one considers a broader spectrum of biological conditions, appropriate grouping samples is non-trivial. It is known that transcriptomes are highly variable across individuals and biological conditions. Therefore, the traditional tissue stratification strategy has hindered the generalization of human UEG studies to a larger scale. As UEGs should be broadly expressed in all tissue/cell types of an organism, we proposed to use a global expression specificity definition based on the proportion of a gene present among diverse transcriptomes (Figure 1). This definition does not require defining discrete tissue/cell type groups and is suitable for dealing with a large collection of transcriptomes. Global exprssion specificity (ϕ) for a gene is defined as:

(2) $$\;\phi =\;\frac{\mathrm{Number}\;\mathrm{of}\;\mathrm{transcriptomes}\;\mathrm{expressing}\;\mathrm{this}\;\mathrm{gene}}{\;\mathrm{Number}\;\mathrm{of}\;\mathrm{total}\;\mathrm{transcritpomes}}$$

The value of global expression specificity ϕ ranges between 1 (for UEGs) and close to 0 (for SEGs).

### Expression detection thresholds

With the definition of global expression specificity, the key problem is the appropriate selection of an expression detection threshold to call whether a gene is expressed. We note that there are different methods to define a detection threshold to call a gene expressed [17]. With those lowly expressed UEG clusters, we compared four detection thresholds, with TPM ≥ 0.1 (which was used in the GTEx project), TPM ≥ 1.0, RPKM ≥ 0.3 [8], [54], [55], and RPKM ≥1.0 [56], [57], [58]. When using the threshold of TPM ≥ 0.1, the median detection rates of lowly expressed cluster #4 genes in the recount2 and DEE2 datasets were 0.83 and 0.89, respectively. However, the detection rates with the threshold of RPKM ≥ 0.3 in the recount2 and DEE2 datasets were only 0.65 and 0.58, respectively (Figures S6 and S20; Table S4). We also observed that the detection sensitivity of the RPKM ≥ 0.3 was close to that of TPM ≥ 1.0. The distribution curves of transcriptome size obtained by RPKM ≥ 0.3 and TPM ≥ 1.0 showed a significant overlap (Figure 3B). Altogether, among these commonly used detection thresholds, the detection threshold of TPM ≥ 0.1, which was used in the GTEx project, was most sensitive for lowly expressed genes and was more appropriate as the expression detection threshold in this study.

### Gene functional annotation and pathway enrichment

The functional gene sets were downloaded from their original publications, and all gene IDs were converted to Entrez IDs by Ensembl BioMart (Table S10). The gene functional enrichment analyses were conducted by DAVID tool [59].

### Quantile normalization and batch effects

To reduce the batch effect and yield a better estimation of global expression patterns, we used a sample-wise quantile transformation to TPM- or RPKM-normalized expression values for each transcriptome profile [18].

(3) $$Q_{j}\left(x\right)=\left\{\begin{array}{c} \frac{\left|i\in \nu :0<x_{i}\leq x_{j}\right|}{\left|\left\{i\in \nu :0<x_{i}\right\}\right|},\;if\;x_{j}>0\\ 0,\;if\;x_{j}=0 \end{array}\right)$$

The quantile normalization returns a normalized expression value $0\leq Q_{j}\left(x\right)\leq 1$.

After transformation, expression values were replaced by their percentile ranks for each profile. Quantile normalization can eliminate most of the biological and technical variances in expression measurements and result in a semi-quantitative representation for expression levels. We examined the relatedness patterns of quantile-normalized profiles by PCA. As shown in Figure S21, the normalized quantile profiles were highly divergent and reasonably repopulated the entire transcriptome space. We then calculated the within-study differences, within-tissue-group differences, and total differences (Figure S22), and observed that the profiles from the same projects showed relatively higher similarity, but the quantile-normalized data significantly reduced the number of outliers. It implies that the quantile normalization method can remove most, but not necessarily all, of the variance attributed to batch. These results suggest that our analysis pipeline may provide a fairly unbiased characterization of gene expression distributions.

### Global expression distributions of relative expression values

The sample distribution attributes, including the 5% percentile (Q5) to the 95% percentile (Q95), the IQR, and the distribution skewness, of relative expression values, were calculated using R. The series of percentile ranks of relative expression values for each gene (Q5 to Q95), *i.e.*, the dynamic range, represent the global expression pattern for each gene. In this study, we used the IQR of distribution to represent the expression variability for each gene. Skewness refers to the asymmetry in expression levels. Negative skewness indicates that most of the data points (genes) are towards the high expression end.

(4) $$\mathrm{Skewness}=\frac{n}{\left(n-1)\times (n-2\right)}\times \sum {\left(\frac{x-\bar{x}}{s}\right)}^{3}$$

where *x* is the relative expression value, $\overset{¯}{x}$ is the sample mean, *s* is the sample standard deviation, and *n* is the number of samples.

### Percentile clustering on dynamic ranges of gene expression level

Our goal is to reduce the dimension of the global expression matrix and cluster genes based on their global expression patterns, where members of the same cluster share similar expression levels, expression variability, and expression specificity (Figure 5). This problem can be formulated as clustering genes by their shapes of distributions. The main difficulty here is the representation of distribution shapes. In this study, we adopted a simple strategy called percentile clustering [22], which uses a series of percentiles of the relative expression values, *i.e.*, the dynamic range matrix, to represent the shape of the distribution, and then uses this percentile matrix to cluster genes. With this strategy, we clustered human genes using an affinity propagation clustering method (APCluster with negDistMat similarity matrices) [60], with the dynamic range matrix. APCluster can infer the number of clusters automatically and provide a representative gene as the local center for each cluster. Our sensitivity analysis showed that the affinity propagation clustering method yielded a better within-cluster homogeneity than the *K*-means method (Figure S23). Figure 5 illustrates the clustering results, and Table S11 lists the gene clusters.

## Code availability

Code is publicly available at https://github.com/macroant/HumanUEGs.

## Competing interests

The authors declare that they have no conflicts of interest.

## CRediT authorship contribution statement

**Jianlei Gu:** Conceptualization, Methodology, Formal analysis, Writing – original draft. **Jiawei Dai:** Data curation, Validation. **Hui Lu:** Supervision, Writing – review & editing, Funding acquisition. **Hongyu Zhao:** Supervision, Writing – review & editing. All authors have read and approved the final manuscript.

## Supplementary material

Supplementary File S1 **Supplementary results**.

Supplementary Figure S1 **The overrepresented level among analyzed transcriptomes**. PCA ordination density plot of the first and second principal components. The color of each hexagon represents the corresponding sample density. The dashed area indicates these overrepresented samples.

Supplementary Figure S2 **Estimate the impacts of overrepresented samples on global expression specificity**. The left figure is the scatter plot of the global expression specificity obtained from total informative samples of the recount2 dataset and the samples which excluded the overrepresented samples. They are highly concordant with each other (pearson coefficient is 0.99). The right figure is the cumulative distribution curve of the absolute differences of the global expression specificity between them and about 90.72% of total genes, have the difference of global expression specificity was less than 0.1 (10% of total range), and the maximal difference is 0.23. The global expression specificity is determined by the threshold of TPM 0.1. TPM.

Supplementary Figure S3 **Estimate the impacts of overrepresented samples on global distribution attributes**. These figures show the four major distribution attributes between total informative samples of the recount2 dataset and the samples which excluded the overrepresented samples. We observed that the overrepresented samples only have a slightly larger impact on the lower bound (Q5) of the distribution of relative expression values.

Supplementary Figure S4 **The differences in distribution attributes between all samples and excluded overrepresented samples**. These figures show the differences of 4 major distribution attributes between total informative samples of the recount2 dataset and the samples which excluded the overrepresented samples. We observed that about 16.90% of the genes had differences larger than 0.1 in Q5. For median relative expression level (Q50), a maximal relative expression level (Q95), and expression variability (IQR), more than 90% of the genes had differences less than 0.1.

Supplementary Figure S5 **Phenotypic compositions of DEE2 samples**. The onlinePCA was performed to the quantile normalized TPM data to visualize the phenotypic compositions and relatedness among transcriptomes for the DEE2 dataset. Each dot represents one transcriptome projected on the principal plane formed by the first and second principal axes. A. The colored dots represent the manually curated reference transcriptomes belonging to 101 tissue groups. Dark dots represent those unclassified transcriptomes that exhibited a broad spectrum of heterogeneity. B. The blue dots are the transcriptomes from tissue samples, and the red dots are the transcriptomes from in vitro cells.

Supplementary Figure S6 **The dynamic ranges of TPM values of LoVarUEGs by gene clusters**. We checked the dynamic ranges of raw TPM values for some LoVarUEGs gene clusters in both recount2 and DEE2 datasets, thus confirming their ubiquitous and stable expression patterns. Dashed lines in the upper two figures are the threshold of TPM 1.0, and solid lines are the threshold of TPM 0.1. Dashed lines in the lower two figures are the threshold of RPKM 1.0, and solid lines are the threshold of RPKM 0.3.

Supplementary Figure S7 **Global expression specificity and tissue specificity of expression**. The left figure is the comparison between global expression specificity and traditional tissue specificity of expression. The right figure is the cumulative distribution curve of the absolute differences between these two metrics. The expression detection threshold is TPM 0.1. We observed that the global expression specificity is highly concordant with traditional tissue specificity of expression (Pearson Coefficients is 0.960) and only 2279 genes (9.1%) that have relatively high divergence (>=0.2) between these two metrics.

Supplementary Figure S8 **2D-density plot between global expression specificity and tissue specificity of expression**. The 2D density plot shows that the genes with higher or lower global expression specificity have a higher agreement between global expression specificity and traditional tissue specificity of expression.

Supplementary Figure S9 **2D-density plot to threshold determination for quantile normalized relative expression values**. The dashed red line is the relative expression level at 0.1 that could be used to empirically distinguish expression genes from non-expressed genes.

Supplementary Figure S10 **Evaluating the global expression patterns for putative disallowed genes of the islets beta cells in DEE2 dataset.** The global expression specificity and global expression patterns of the 16 putative disallowed genes are consistent with the observations in the recount2 dataset.

Supplementary Figure S11 **Comparison of standard deviations of TPM values between LoVarUEGs and 2013HK**. Compared with the previously reported HK genes with stable expression, we observed that they have comparable standard deviations of expression in both recount2 and DEE2 datasets. The LoVarUEGs is provided in Table S4, and their dynamic ranges are provided in Tables S8 and S9.

Supplementary Figure S12 **Comparison of coefficient of variations of TPM values between LoVarUEGs and 2013HK**. Compared with the previously reported HK genes with stable expression, we observed that they have comparable coefficient of variations of expression in both recount2 and DEE2 datasets.

Supplementary Figure S13 **Comparison between single-cell expression stability and bulk expression specificity**. We mapped the expression stability of the single-cell onto our gene clusters and observed that the sparsity (fraction of zeros) of single-cell profiles highly correlates with the global expression specificity and the expression level at bulk level. Left figure is all 96 gene clusters ranked by their median global expression specificity. Right figure is the 19 UEGs clusters with low expression variability, including cluster #81, #13, #25, #67, #28, #75, #33, #91, #74, #34, #85, #2, #10, #43, #12, #62, #87, #77, and #4, and 9 MEGs/SEGs clusters with low expression variability #70, #48, #39, #54, #23, #14, #32, #37, and #86. (From left to right in the right figure).

Supplementary Figure S14 **The reproducibility of global expression specificity between two separate datasets**. The left figure is the scatter plot of the global expression specificity obtained from recount2 and DEE2, respectively. The right figure is the cumulative distribution curve of the absolute differences in the global expression specificity between these two datasets. The global expression specificity is determined by the threshold of TPM 0.1.

Supplementary Figure S15 **The reproducibility of global distribution attributes of relative expression values between two separate datasets**. These figures show the correlations of 4 major distribution attributes between recount2 and DEE2 datasets. We observed that they are highly concordant with each other.

Supplementary Figure S16 **The differences in distribution attributes between recount2 and DEE2 samples**. These figures show the differences of 4 major distribution attributes between recount2 and DEE2 samples. We observed that about 16.61 % of the genes had differences larger than 0.2 in Q5. For median relative expression level (Q50), a maximal relative expression level (Q95), and expression variability (IQR), more than 90% of the genes had their difference less than 0.2.

Supplementary Figure S17 **Comparison of UEGs with previous studies**. Comparisons with previous UEGs and SEGs studies (Table 1) showed that (1) early microarray-based UEG studies significantly underestimated the number of human UEGs; (2) Over 95% of previously reported UEGs were validated in our study (ϕ >= 0.8); (3) We identified 2804 novel UEGs, 73.57% of which were also found in the separate dataset DEE2; (4) A total of 86.2% of UEGs generated from recount2 and DEE2 overlapped.

Supplementary Figure S18 **The SEGs identified by the identical method but different datasets exhibited significant discrepancy**. Even using the same method, the specifically expressed genes identified by different datasets still showed considerable levels of discrepancy. These two gene sets were downloaded from a recent SEGs study [3].

Supplementary Figure S19 **Transcriptome profile quality control for Recount2 dataset**. A transcriptome was considered as a low-quality profile if any of 3 low-expression internal reference genes (GUSB, HPRT1, and HMBS) had expression measurements of zero and was eliminated for further analyses. We used a boxplot to compare the sparsity level between the low-quality profiles and the informative profiles.

Supplementary Figure S20 **Comparison of the global expression specificity categories identified by different normalization methods and datasets**. We compared the global expression categories between different expression detection thresholds, including TPM 0.1, TPM 1.0, RPKM 0.3, and RPKM 1.0. The global expression patterns and gene clusters were obtained from recount2 quantile normalized TPM data.

Supplementary Figure S21 **Evaluating the batch effects of transcriptome profiles, onlinePCA comparison**. After quantile normalization, we observed that the data points (recount2 samples) reasonably repopulate the entire transcriptome space. It implies the quantile normalized data significantly reduced the batch effects.

Supplementary Figure S22 **Evaluating the batch effects of transcriptome profiles, compare within-group similarity**. We randomly selected 5000 profiles and calculated the euclidean distance between data points that within-tissue-group, within-projects, and total background. We observed that the profiles from the same projects show relatively higher similarity, but the quantile normalized data significantly reduced the number of outliers. It implies that the quantile normalization method can remove most, but not necessarily all, of the variance attributed to batch.

Supplementary Figure S23 **Comparison of within-cluster homogeneity between affinity propagation and k-mean method**. The left figure is the clustering results of the k-means method with the sample cluster number (96) of the AP method. The right figure shows that the AP method yielded better within-cluster homogeneity than the K-means method. AP, affinity propagation.

Supplementary Figure S24 **Estimate the impacts of sample size on global expression specificity**. We conducted 100 times random sampling for each gene and a series of sampling sizes (x-axis), including 1000, 5000, 10000, 15000, 20000, 25000, 25000, 30000, and 35000. The global expression specificity becomes stable as the sampling size approaches 35,000. The median maximal difference of global expression specificity is 0.0027 (0.25% of total range).

Supplementary Figure S25 **Estimate the impacts of sample size on global distribution attributes**. We conducted 100 times random sampling for each gene and sampling size (x-axis), including 1000, 5000, 10000, 15000, 20000, 25000, 25000, 30000, and 35000. The 4 major distribution attributes become stable as the sampling size approaches 35,000. The median maximal difference of these attributes is less than 0.004 (0.4% of total range).

Supplementary Figure S26 **Evaluation of the number of clusters by gap statistics**. The quantile normalized TPM data with K-means clustering method was used to calculate this gap statistics. Generally, cluster number with maximum gap statistic value corresponds to the optimal number of clusters. As k increases, the gap statistics show continuous and smooth growth. However, we did not observe a clear elbow point.
