# Supplementary material for: Comprehensive Analysis of Ubiquitously Expressed Genes in Humans from A Data-driven Perspective
Source: Genomics Proteomics Bioinformatics. 2022 May 13;21(1):164–76. doi: 10.1016/j.gpb.2021.08.017 (PMC10373092; doi:10.1016/j.gpb.2021.08.017)
Supplement: Supplementary File S1 — Supplementary results. [file mmc1.docx]

### **File S1 Supplementary results**

### **Sensitivity analysis for global expression specificity and global distribution attributes**

As shown in Figure S1, we determined the overrepresented sample by a 2-dimensional (2D) density plot of the first two principal components of onlinePCA [1]. We first checked the phenotypic compositions of these overrepresented samples and observed that they are high divergent in phenotypic compositions (Tables S12 and S13). To test if these overrepresented samples would affect global expression specificity and the distribution attributes significantly, we excluded those overrepresented samples and compared their global expression specificity and global distribution attributes with that of all samples. Figure S2 shows that ~90.72% of the genes had differences of global expression specificity less than 0.1 (10% of total range), and the maximal difference was 0.23. For the global distribution attributes (Figures S3 and S4), the overrepresented samples had a slightly larger impact on the lower bound (Q5, 5% percentile) of the distribution of relative expression values, and 16.90% of the genes had their difference larger than 0.1 in Q5. For median relative expression level (Q50, 50% percentile), a maximal relative expression level (Q95, 95% percentile), and expression variability (IQR, Interquartile range), more than 90% of the genes had their difference less than 0.1. Collectively, these overrepresented samples had limited effects on the final global expression specificity and distribution attributes. To retain as much information, we used all informative transcriptomes from the recount2 and DEE2 datasets.

Moreover, to evaluate the effects of sample size on the results, we conducted 100 random sampling with a series of sampling sizes for each gene. As shown in Figures S24 and S25, the global expression specificity and distribution attributes became stable as the sample size approached 35,000. These results indicate that we can estimate global expression specificity and global distribution attributes of each gene with a high degree of confidence with a large number of the transcriptomes.

### **Sensitivity analysis for percentile clustering**

To validate the clustering robustness and to provide more stable clustering results, we conducted a sensitivity analysis for our percentile clustering results. We assume that the putative variations in clustering results could be largely attributed to (1) the variations in the dynamic range matrix, *i.e.*, the global distribution attributes; (2) The number of clusters; And/or (3) the clustering method.

For the first question, we have discussed in the sensitivity analysis. The results showed that the dynamic range matrix, *i.e.*, global distribution attributes, is highly stable and robust. For the second question, we observed that the dynamic ranges of gene expression are continuous (Figure 3A), and it means that there does not seem to be a clear separation boundary in terms of global expression patterns among human genes. We then used the gap-statistic to show the clustering tendency of global expression patterns. The gap statistic compares the sum of within-cluster variations for different numbers of k with their expected values under the null reference distribution of the data. Generally, cluster number with maximum gap statistic value, which is an elbow point, corresponds to the optimal number of clusters. As shown in Figure S26, as k increased, the gap statistic showed a continuous increase that means the expression patterns do not have a clear optimal number of clusters. To determine the optimal number of clusters or cluster boundaries, we used the affinity propagation clustering method, which can automatically select the optimal number of clusters and does not require the number of clusters to be specified in advance. By comparing clustering results on different datasets and normalization methods, the number of clusters is around 90−96. In this study, we used the clustering results from the recount2 dataset (Table S11, the number of clusters is 96). For the third question, the affinity propagation clustering method is a non-hierarchical clustering method, which simultaneously considers all data points as potential local centers (exemplar), and it requires that all data points within a cluster be similar to its local center (exemplar) [2,3]. As shown in Figure S23, we made a comparison between affinity propagation and the k-means method using the same cluster number and observed that the affinity propagation method yielded better within-cluster homogeneity (euclidean distance) than the k-means method.

Collectively, clustering genes by their global expression patterns can better group genes into local homogeneous groups that have similar expression patterns, *e.g.*, expression level, expression variability, and expression specificity. To determine the robustness of clustering results, we validated the expression patterns and global expression specificity of the three clusters in an independent dataset, DEE2. We observed that the gene clusters identified in the recount2 data showed similar expression patterns in the DEE2 dataset (Figure S6). We further compared the UEGs (ubiquitously expressed genes) categories identified by gene clusters and observed that the UEGs category is highly reproducible between the recount2 and DEE2 datasets (86.2% overlapping, Figure S17). These results suggest the effectiveness and robustness of the percentile clustering strategy in this study.

## **References**

[1] Degras D, Cardot H. R package: Online Principal Component Analysis. 1.3.1 ed. https://cran.r-project.org/package=onlinePCA: CRAN; 2016.

[2] Frey BJ, Dueck D. Clustering by passing messages between data points. Science 2007;315:972–6.

[3] Bodenhofer U, Kothmeier A, Hochreiter S. APCluster: an R package for affinity propagation clustering. Bioinformatics 2011;27:2463–4.
