# Supplementary material for: Comprehensive Analysis of Ubiquitously Expressed Genes in Humans from A Data-driven Perspective
Source: Genomics Proteomics Bioinformatics. 2022 May 13;21(1):164–76. doi: 10.1016/j.gpb.2021.08.017 (PMC10373092; doi:10.1016/j.gpb.2021.08.017)
Supplement: Supplementary Figure S1 — The overrepresented level among analyzed transcriptomes. PCA ordination density plot of the first and second principal components. The color of each hexagon represents the corresponding sample density. The dashed area indicates these overrepresented samples. [file mmc2.pptx]

## Slide 1
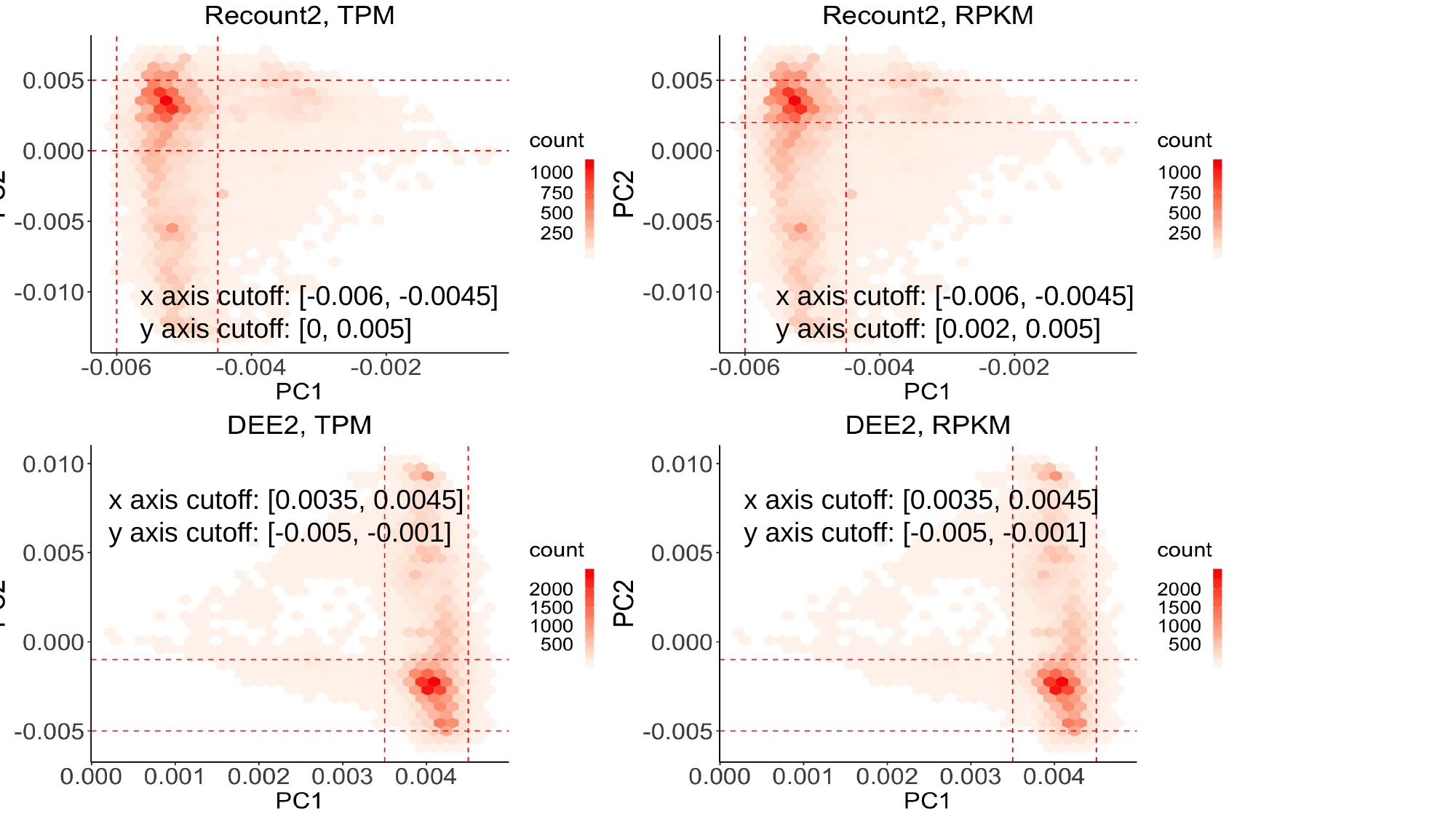

x axis cutoff: [-0.006, -0.0045]
y axis cutoff: [0.002, 0.005]
x axis cutoff: [-0.006, -0.0045]
y axis cutoff: [0, 0.005]
x axis cutoff: [0.0035, 0.0045]
y axis cutoff: [-0.005, -0.001]
x axis cutoff: [0.0035, 0.0045]
y axis cutoff: [-0.005, -0.001]
