# Supplementary figures and images for: Comprehensive Analysis of Ubiquitously Expressed Genes in Humans from A Data-driven Perspective
Source: Genomics Proteomics Bioinformatics. 2022 May 13;21(1):164–76. doi: 10.1016/j.gpb.2021.08.017 (PMC10373092; doi:10.1016/j.gpb.2021.08.017)

## Slide 1
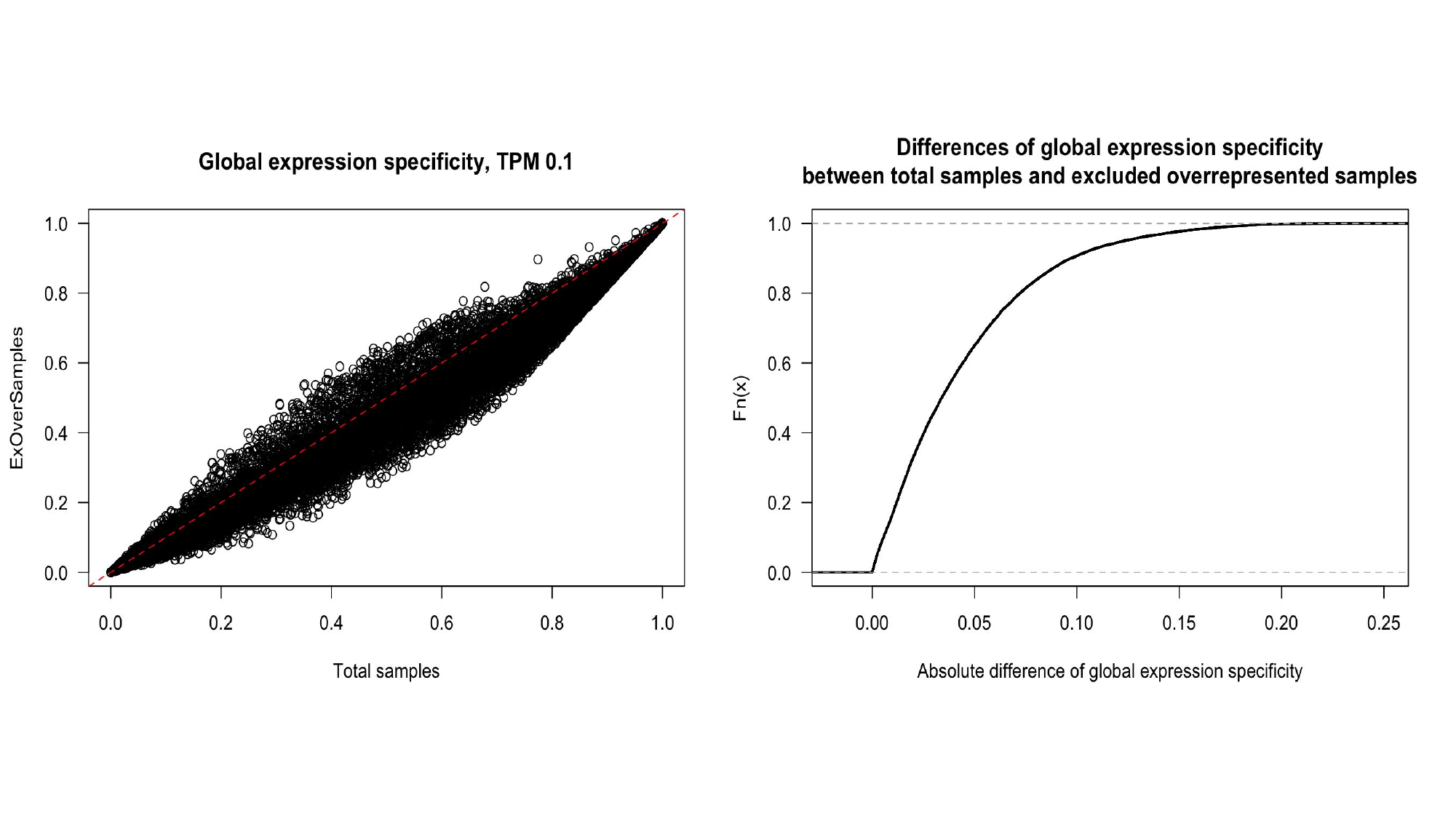

Supplement: Supplementary Figure S2 — Estimate the impacts of overrepresented samples on global expression specificity. The left figure is the scatter plot of the global expression specificity obtained from total informative samples of the recount2 dataset and the samples which excluded the overrepresented samples. They are highly concordant with each other (pearson coefficient is 0.99). The right figure is the cumulative distribution curve of the absolute differences of the global expression specificity between them and about 90.72% of total genes, have the difference of global expression specificity was less than 0.1 (10% of total range), and the maximal difference is 0.23. The global expression specificity is determined by the threshold of TPM 0.1. TPM. [file mmc3.pptx]

## Slide 1
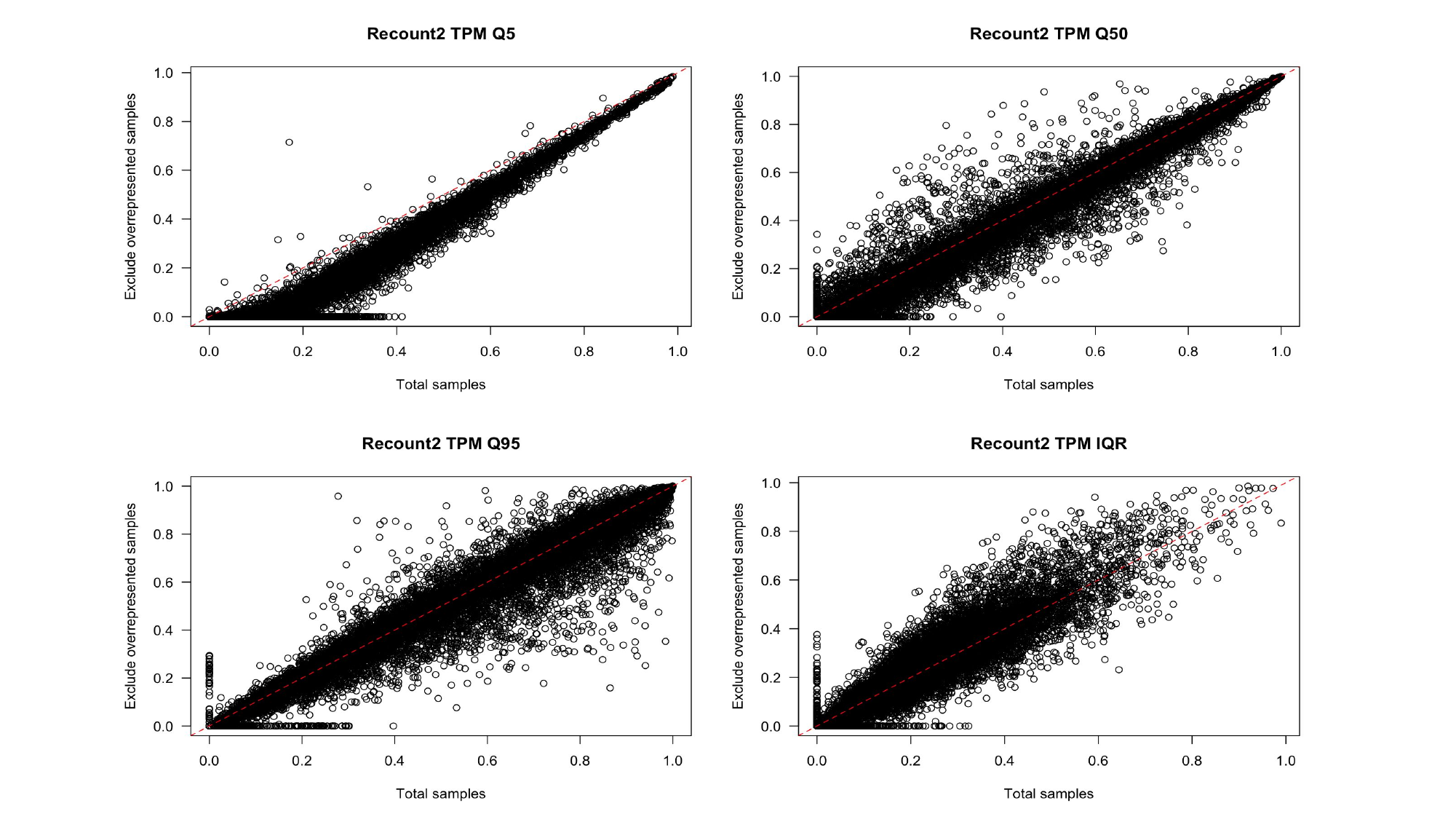

Supplement: Supplementary Figure S3 — Estimate the impacts of overrepresented samples on global distribution attributes. These figures show the four major distribution attributes between total informative samples of the recount2 dataset and the samples which excluded the overrepresented samples. We observed that the overrepresented samples only have a slightly larger impact on the lower bound (Q5) of the distribution of relative expression values. [file mmc4.pptx]

## Slide 1
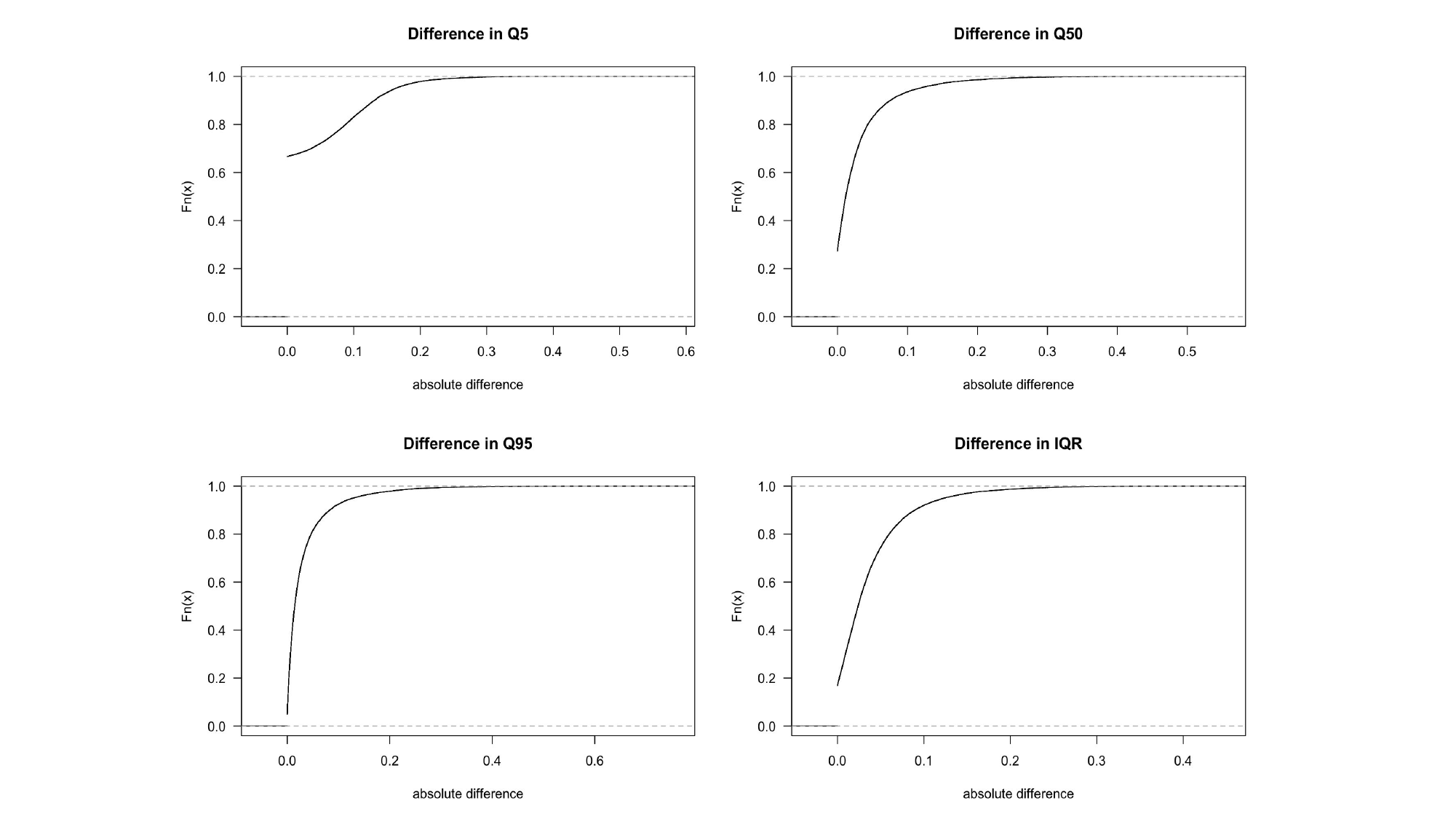

Supplement: Supplementary Figure S4 — The differences in distribution attributes between all samples and excluded overrepresented samples. These figures show the differences of 4 major distribution attributes between total informative samples of the recount2 dataset and the samples which excluded the overrepresented samples. We observed that about 16.90% of the genes had differences larger than 0.1 in Q5. For median relative expression level (Q50), a maximal relative expression level (Q95), and expression variability (IQR), more than 90% of the genes had differences less than 0.1. [file mmc5.pptx]

## Slide 1
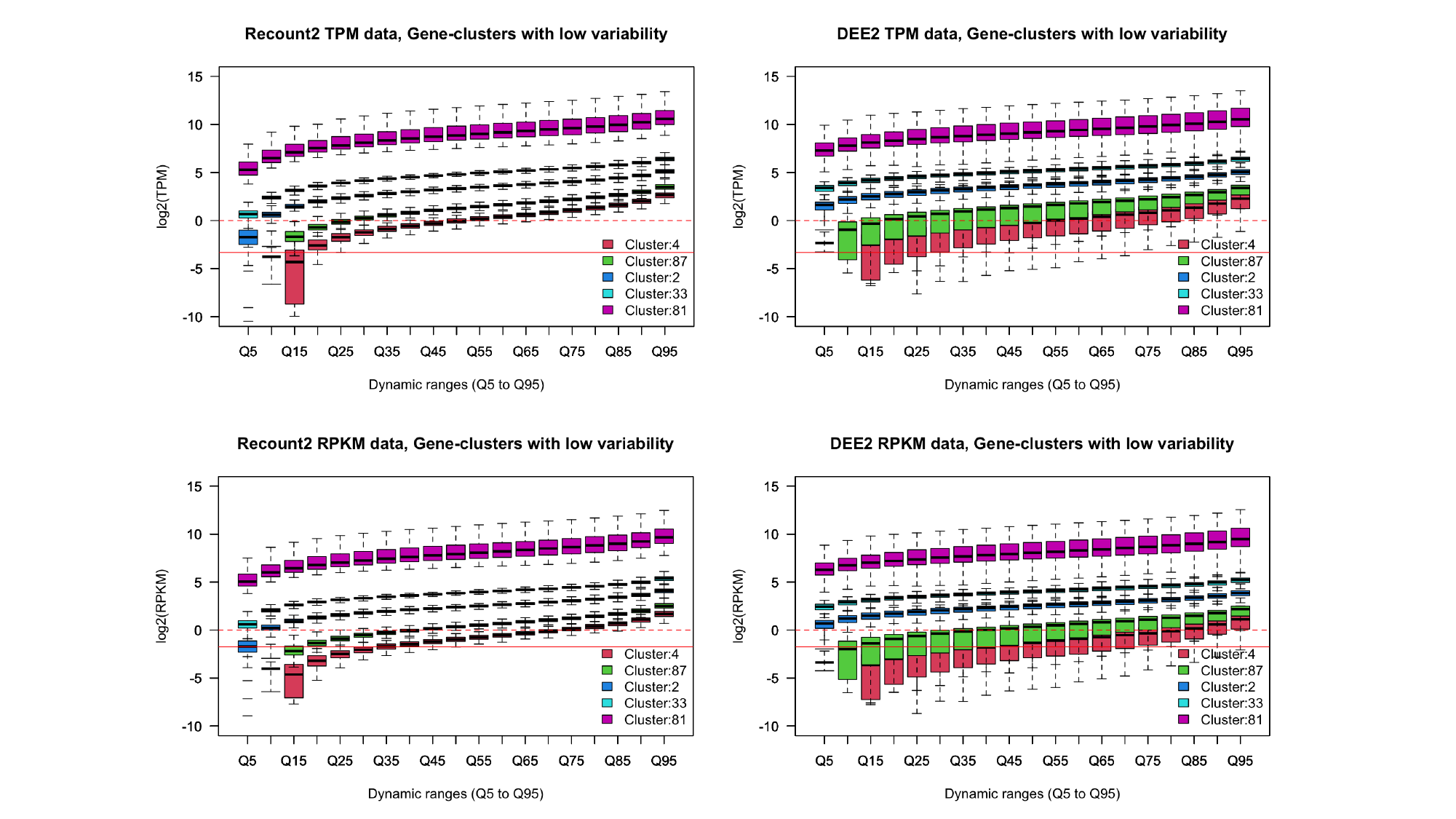

Supplement: Supplementary Figure S6 — The dynamic ranges of TPM values of LoVarUEGs by gene clusters. We checked the dynamic ranges of raw TPM values for some LoVarUEGs gene clusters in both recount2 and DEE2 datasets, thus confirming their ubiquitous and stable expression patterns. Dashed lines in the upper two figures are the threshold of TPM 1.0, and solid lines are the threshold of TPM 0.1. Dashed lines in the lower two figures are the threshold of RPKM 1.0, and solid lines are the threshold of RPKM 0.3. [file mmc7.pptx]

## Slide 1
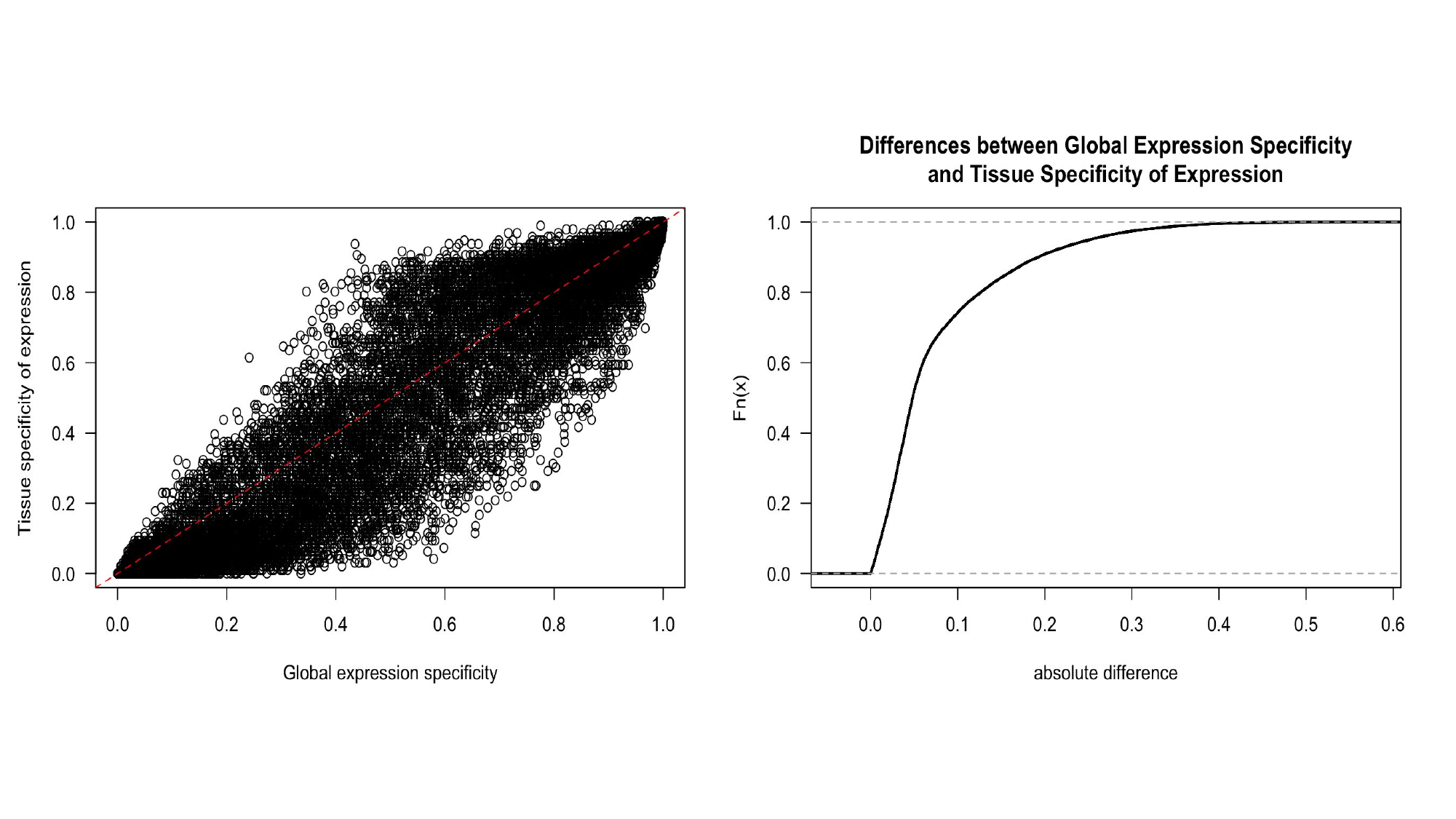

Supplement: Supplementary Figure S7 — Global expression specificity and tissue specificity of expression. The left figure is the comparison between global expression specificity and traditional tissue specificity of expression. The right figure is the cumulative distribution curve of the absolute differences between these two metrics. The expression detection threshold is TPM 0.1. We observed that the global expression specificity is highly concordant with traditional tissue specificity of expression (Pearson Coefficients is 0.960) and only 2279 genes (9.1%) that have relatively high divergence (>=0.2) between these two metrics. [file mmc8.pptx]

## Slide 1
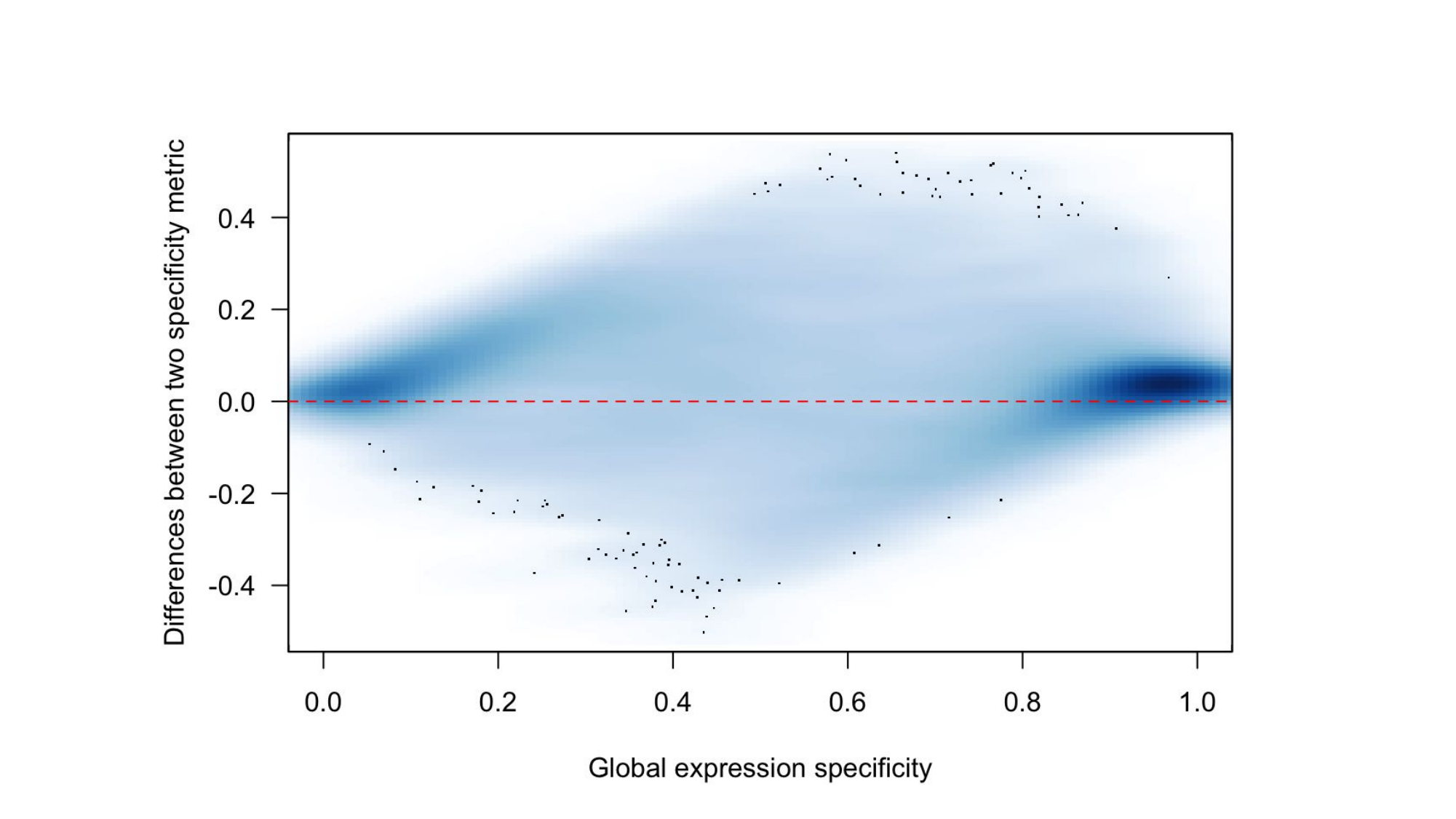

Supplement: Supplementary Figure S8 — 2D-density plot between global expression specificity and tissue specificity of expression. The 2D density plot shows that the genes with higher or lower global expression specificity have a higher agreement between global expression specificity and traditional tissue specificity of expression. [file mmc9.pptx]

## Slide 1
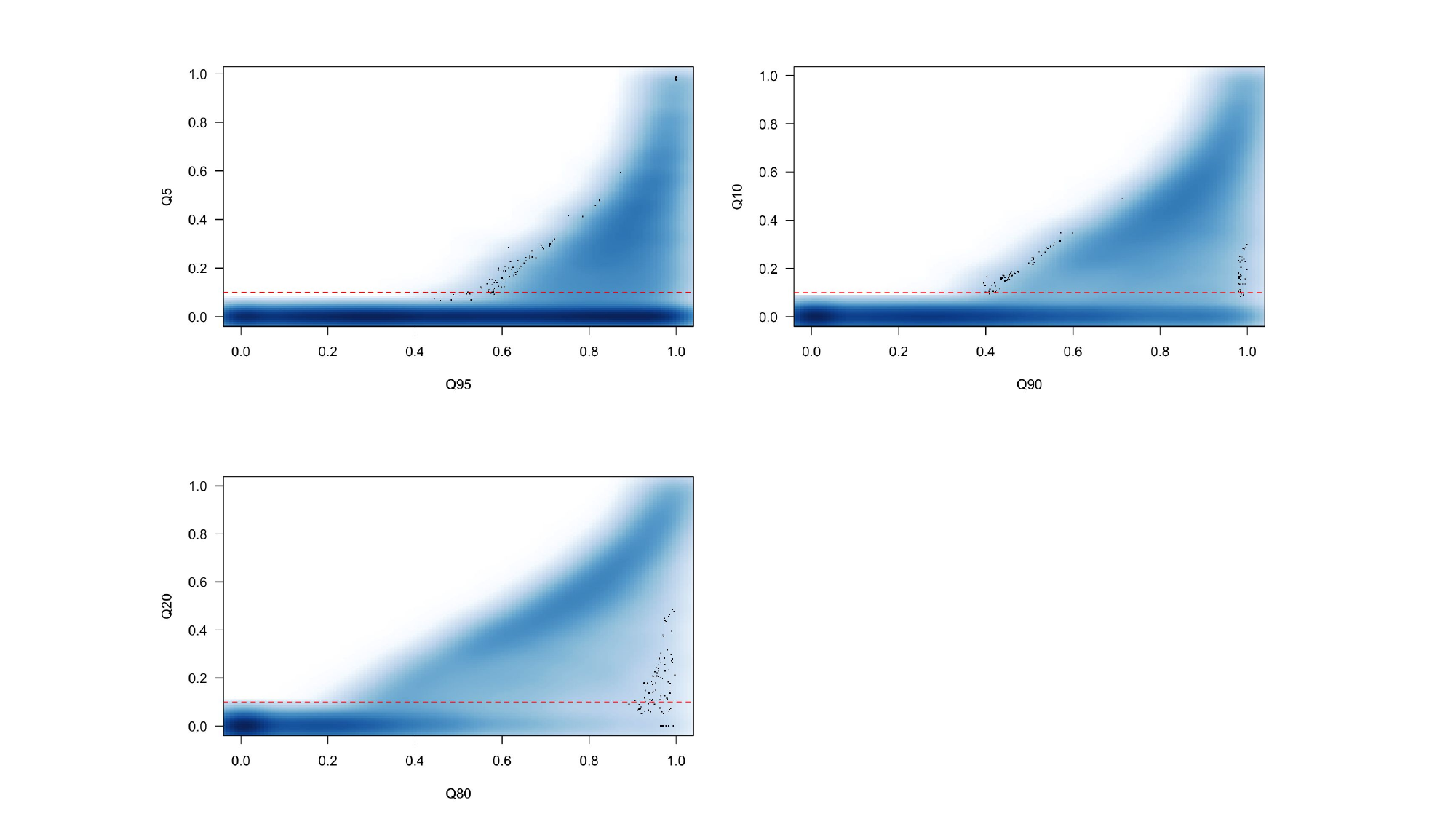

Supplement: Supplementary Figure S9 — 2D-density plot to threshold determination for quantile normalized relative expression values. The dashed red line is the relative expression level at 0.1 that could be used to empirically distinguish expression genes from non-expressed genes. [file mmc10.pptx]

## Slide 1
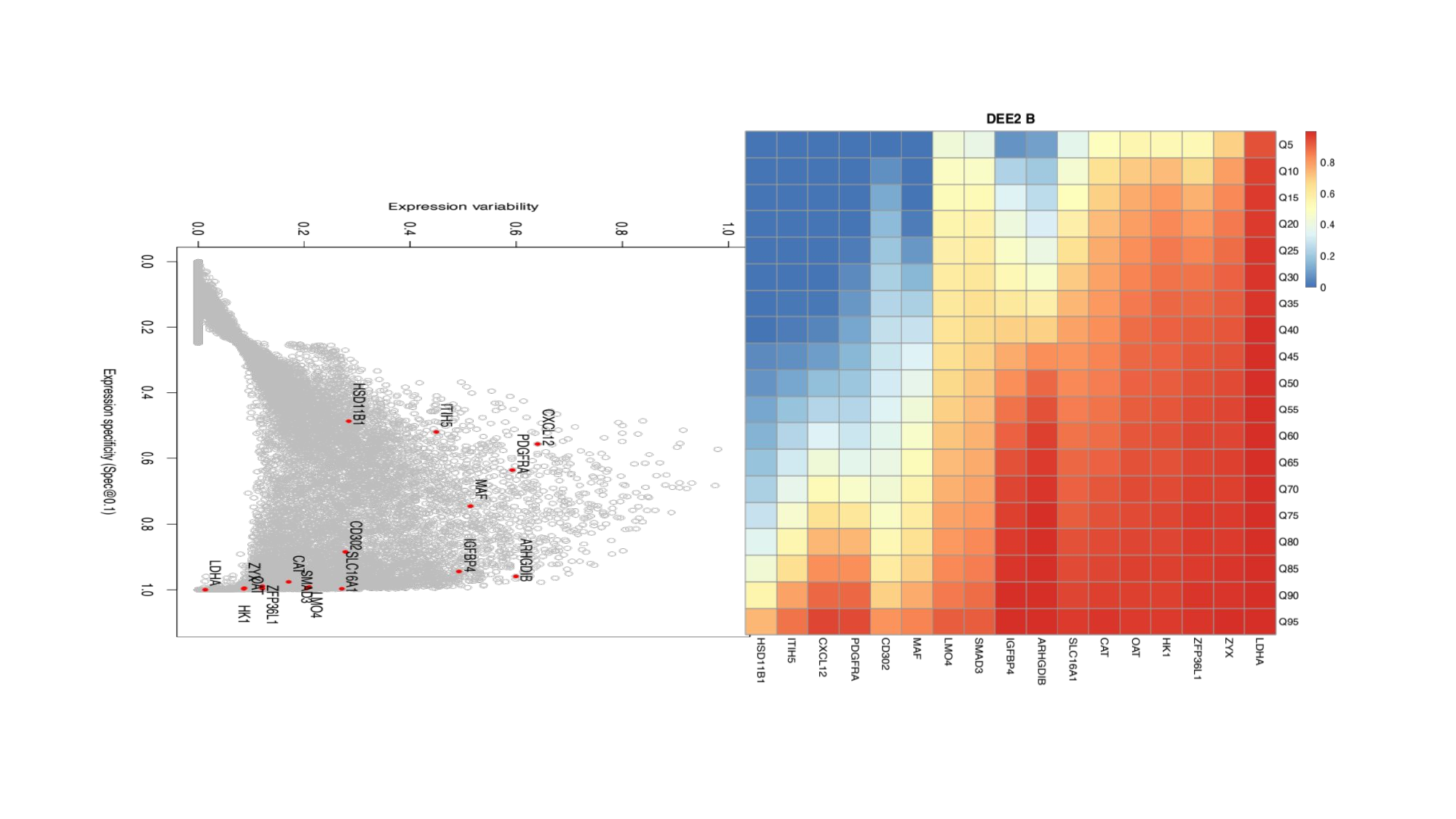

Supplement: Supplementary Figure S10 — Evaluating the global expression patterns for putative disallowed genes of the islets beta cells in DEE2 dataset. The global expression specificity and global expression patterns of the 16 putative disallowed genes are consistent with the observations in the recount2 dataset. [file mmc11.pptx]

## Slide 1
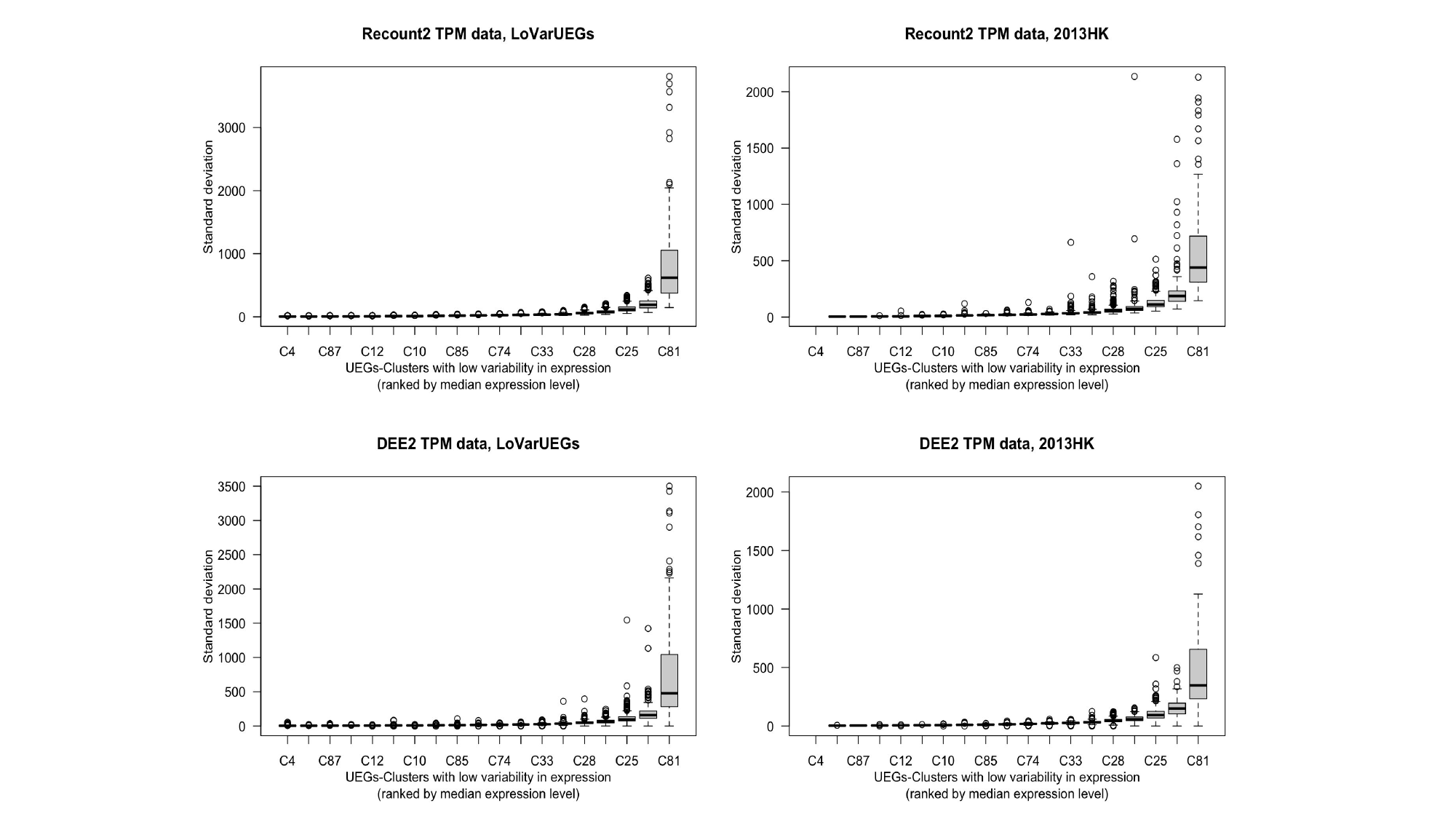

Supplement: Supplementary Figure S11 — Comparison of standard deviations of TPM values between LoVarUEGs and 2013HK. Compared with the previously reported HK genes with stable expression, we observed that they have comparable standard deviations of expression in both recount2 and DEE2 datasets. The LoVarUEGs is provided in Table S4, and their dynamic ranges are provided in Tables S8 and S9. [file mmc12.pptx]

## Slide 1
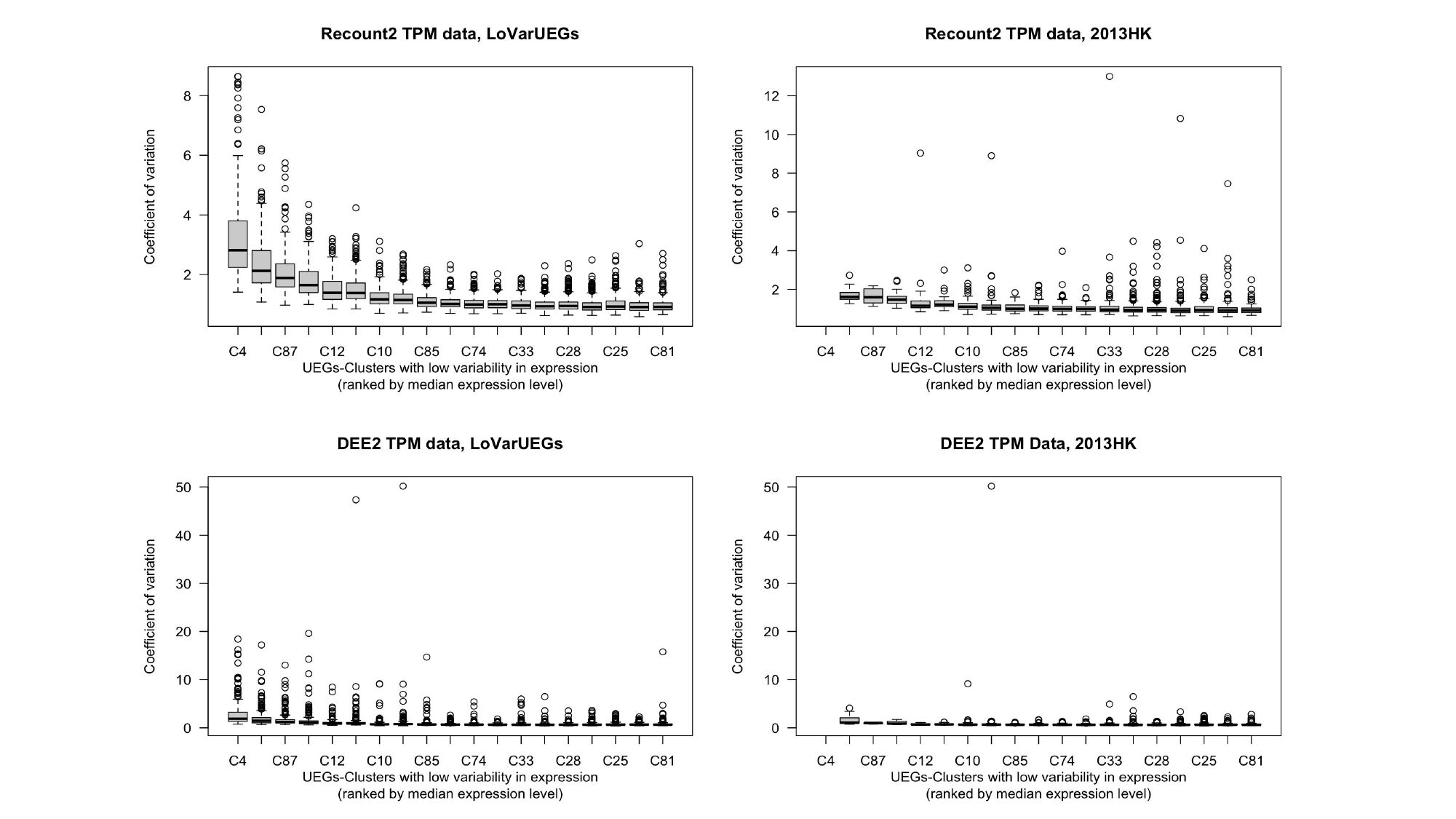

Supplement: Supplementary Figure S12 — Comparison of coefficient of variations of TPM values between LoVarUEGs and 2013HK. Compared with the previously reported HK genes with stable expression, we observed that they have comparable coefficient of variations of expression in both recount2 and DEE2 datasets. [file mmc13.pptx]

## Slide 1
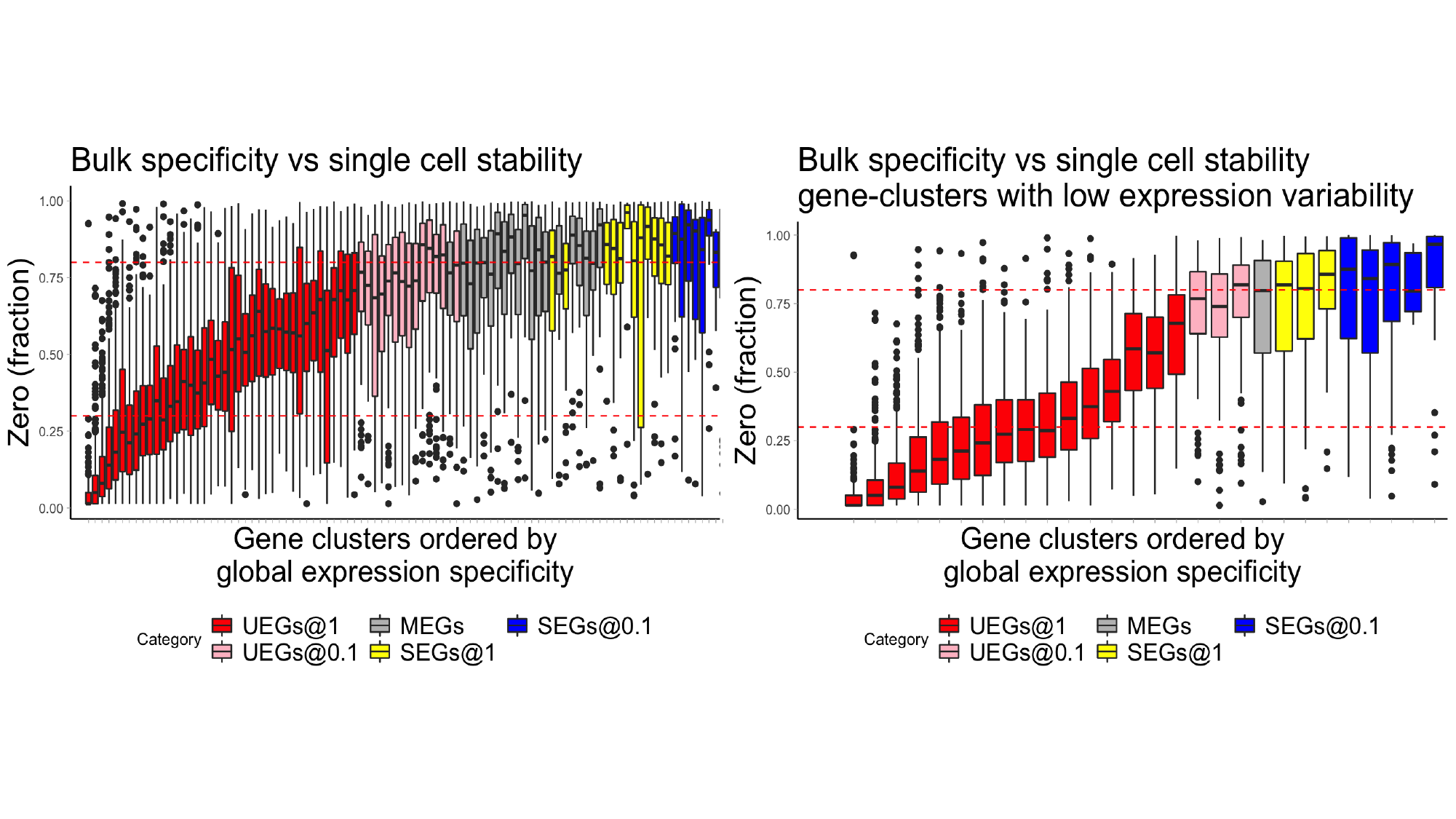

Supplement: Supplementary Figure S13 — Comparison between single-cell expression stability and bulk expression specificity. We mapped the expression stability of the single-cell onto our gene clusters and observed that the sparsity (fraction of zeros) of single-cell profiles highly correlates with the global expression specificity and the expression level at bulk level. Left figure is all 96 gene clusters ranked by their median global expression specificity. Right figure is the 19 UEGs clusters with low expression variability, including cluster #81, #13, #25, #67, #28, #75, #33, #91, #74, #34, #85, #2, #10, #43, #12, #62, #87, #77, and #4, and 9 MEGs/SEGs clusters with low expression variability #70, #48, #39, #54, #23, #14, #32, #37, and #86. (From left to right in the right figure). [file mmc14.pptx]

## Slide 1
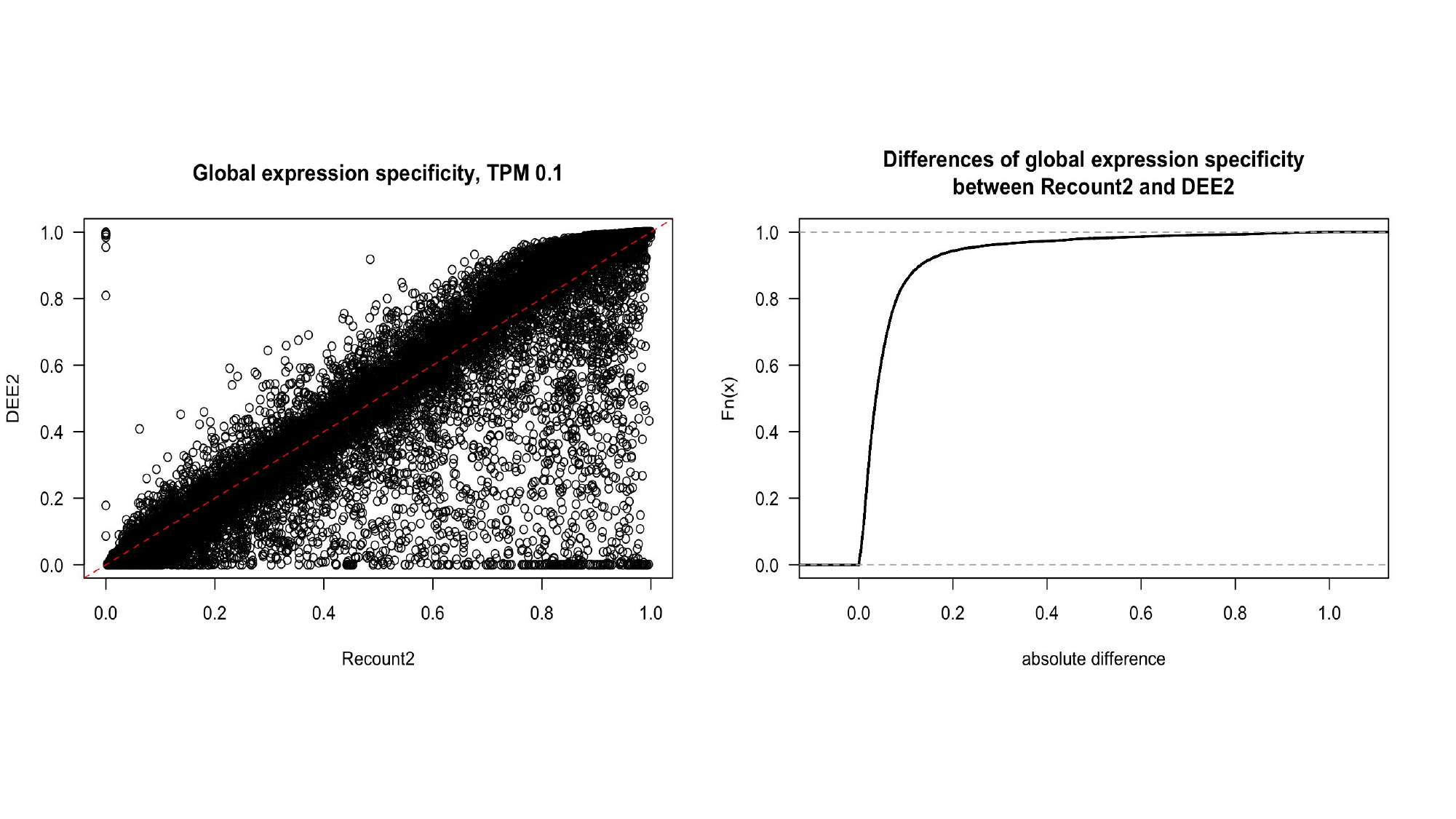

Supplement: Supplementary Figure S14 — The reproducibility of global expression specificity between two separate datasets. The left figure is the scatter plot of the global expression specificity obtained from recount2 and DEE2, respectively. The right figure is the cumulative distribution curve of the absolute differences in the global expression specificity between these two datasets. The global expression specificity is determined by the threshold of TPM 0.1. [file mmc15.pptx]

## Slide 1
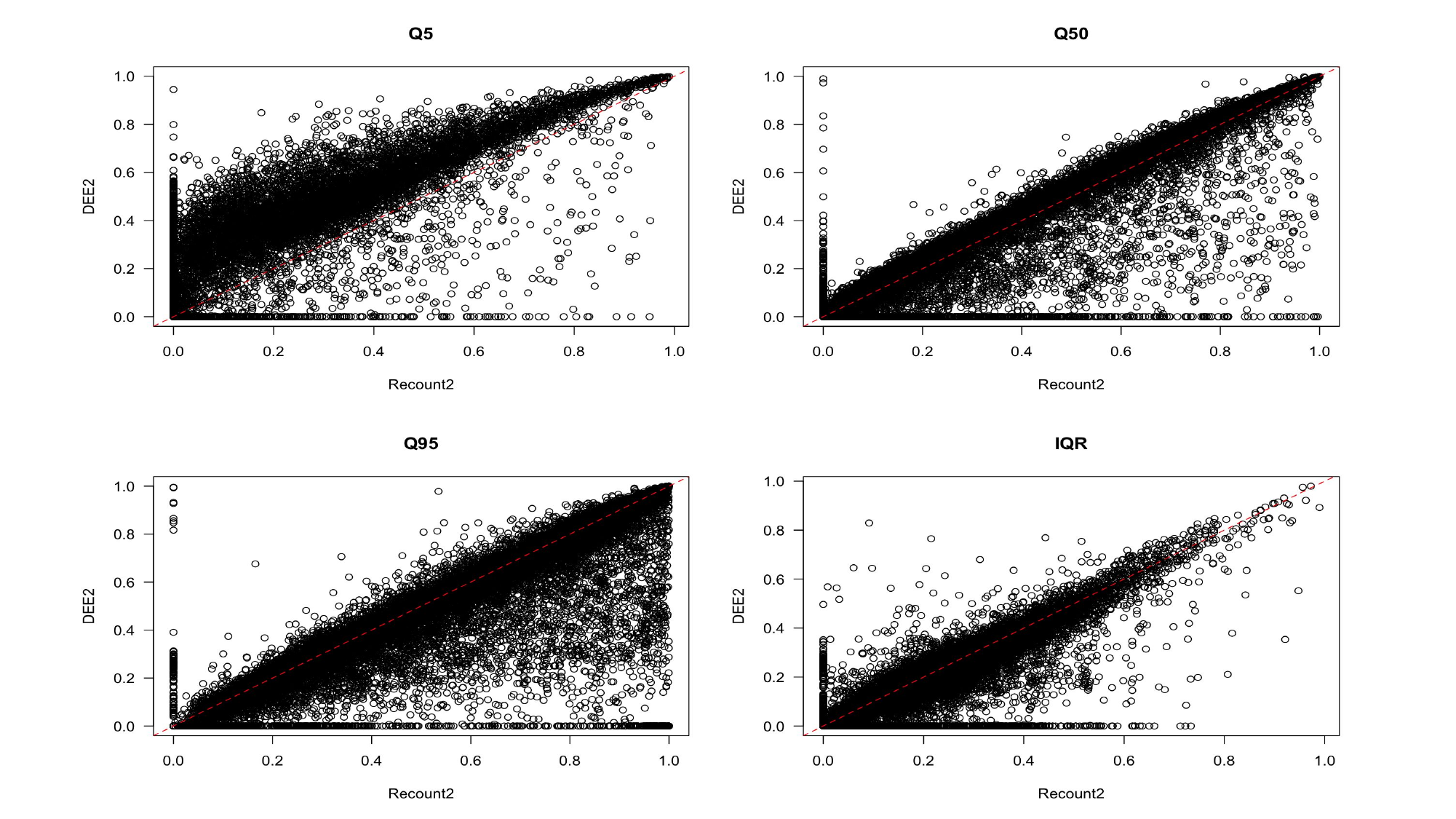

Supplement: Supplementary Figure S15 — The reproducibility of global distribution attributes of relative expression values between two separate datasets. These figures show the correlations of 4 major distribution attributes between recount2 and DEE2 datasets. We observed that they are highly concordant with each other. [file mmc16.pptx]

## Slide 1
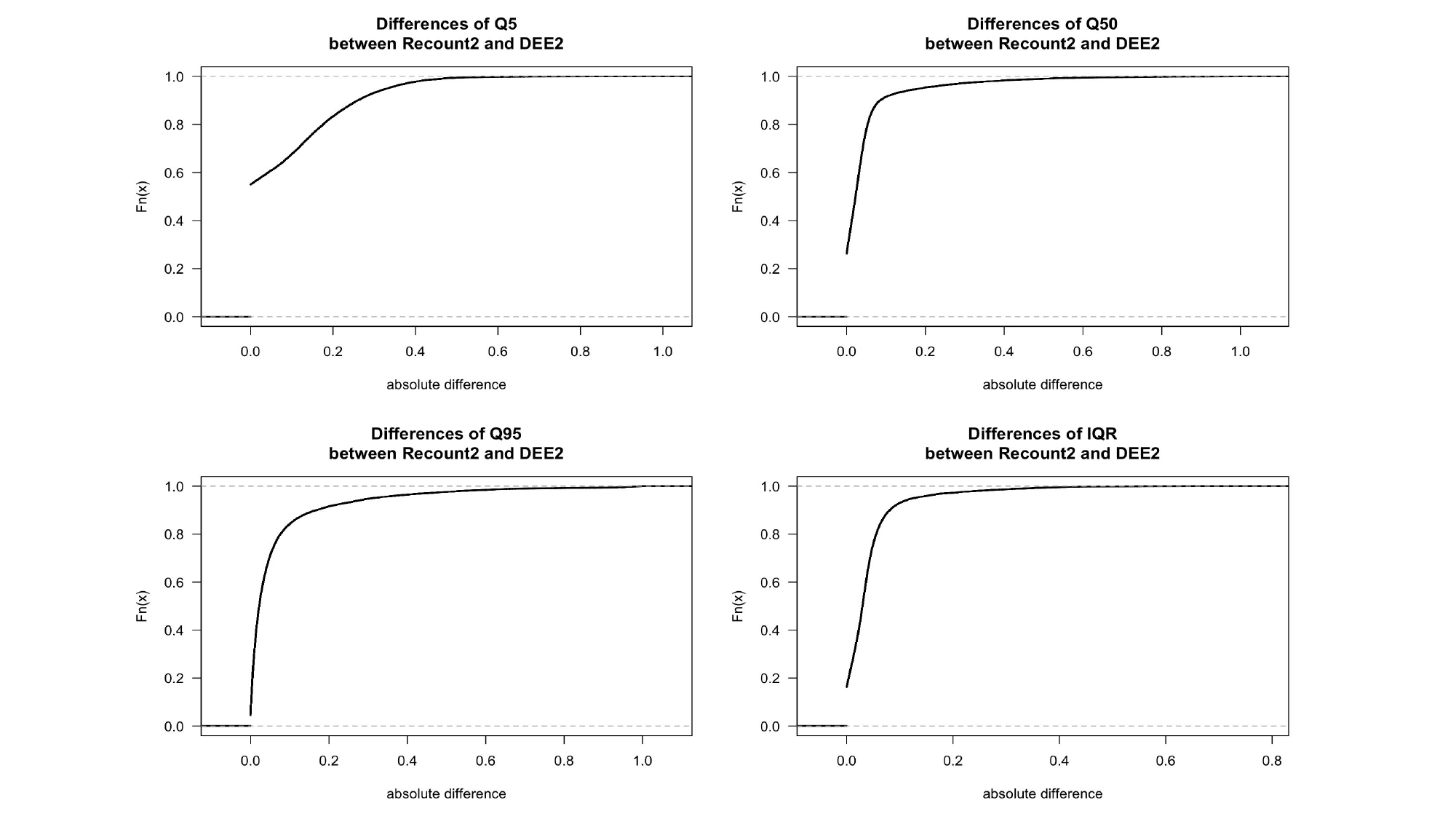

Supplement: Supplementary Figure S16 — The differences in distribution attributes between recount2 and DEE2 samples. These figures show the differences of 4 major distribution attributes between recount2 and DEE2 samples. We observed that about 16.61 % of the genes had differences larger than 0.2 in Q5. For median relative expression level (Q50), a maximal relative expression level (Q95), and expression variability (IQR), more than 90% of the genes had their difference less than 0.2. [file mmc17.pptx]

## Slide 1
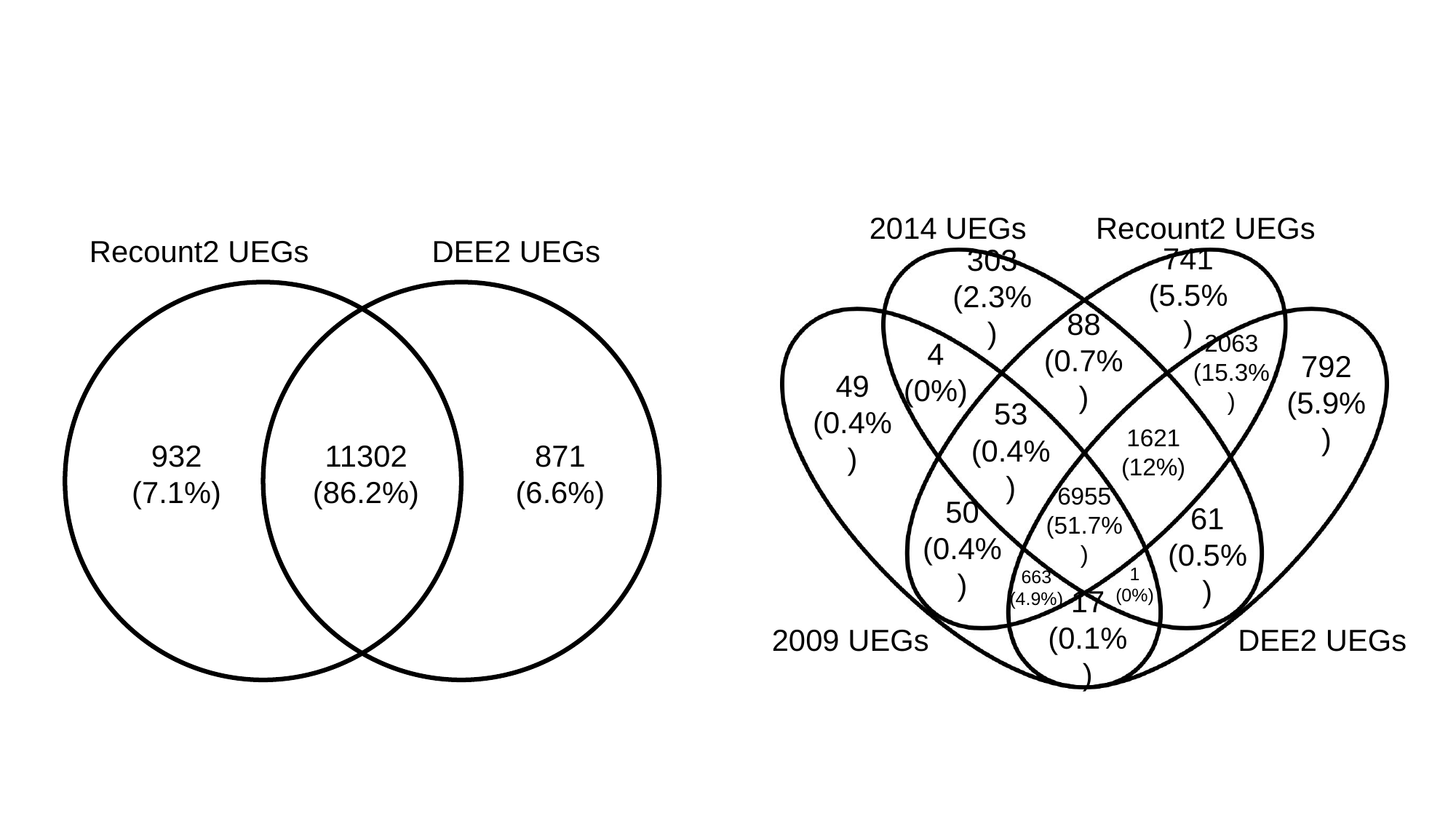

2014 UEGs
Recount2 UEGs
741
(5.5%)
303
(2.3%)
88
(0.7%)
4
(0%)
2063
(15.3%)
792
(5.9%)
49
(0.4%)
53
(0.4%)
1621
(12%)
6955
(51.7%)
50
(0.4%)
61
(0.5%)
1
(0%)
663
(4.9%)
17
(0.1%)
2009 UEGs
DEE2 UEGs
DEE2 UEGs
Recount2 UEGs
871
(6.6%)
11302
(86.2%)
932
(7.1%)

Supplement: Supplementary Figure S17 — Comparison of UEGs with previous studies. Comparisons with previous UEGs and SEGs studies (Table 1) showed that (1) early microarray-based UEG studies significantly underestimated the number of human UEGs; (2) Over 95% of previously reported UEGs were validated in our study (ϕ >= 0.8); (3) We identified 2804 novel UEGs, 73.57% of which were also found in the separate dataset DEE2; (4) A total of 86.2% of UEGs generated from recount2 and DEE2 overlapped. [file mmc18.pptx]

## Slide 1
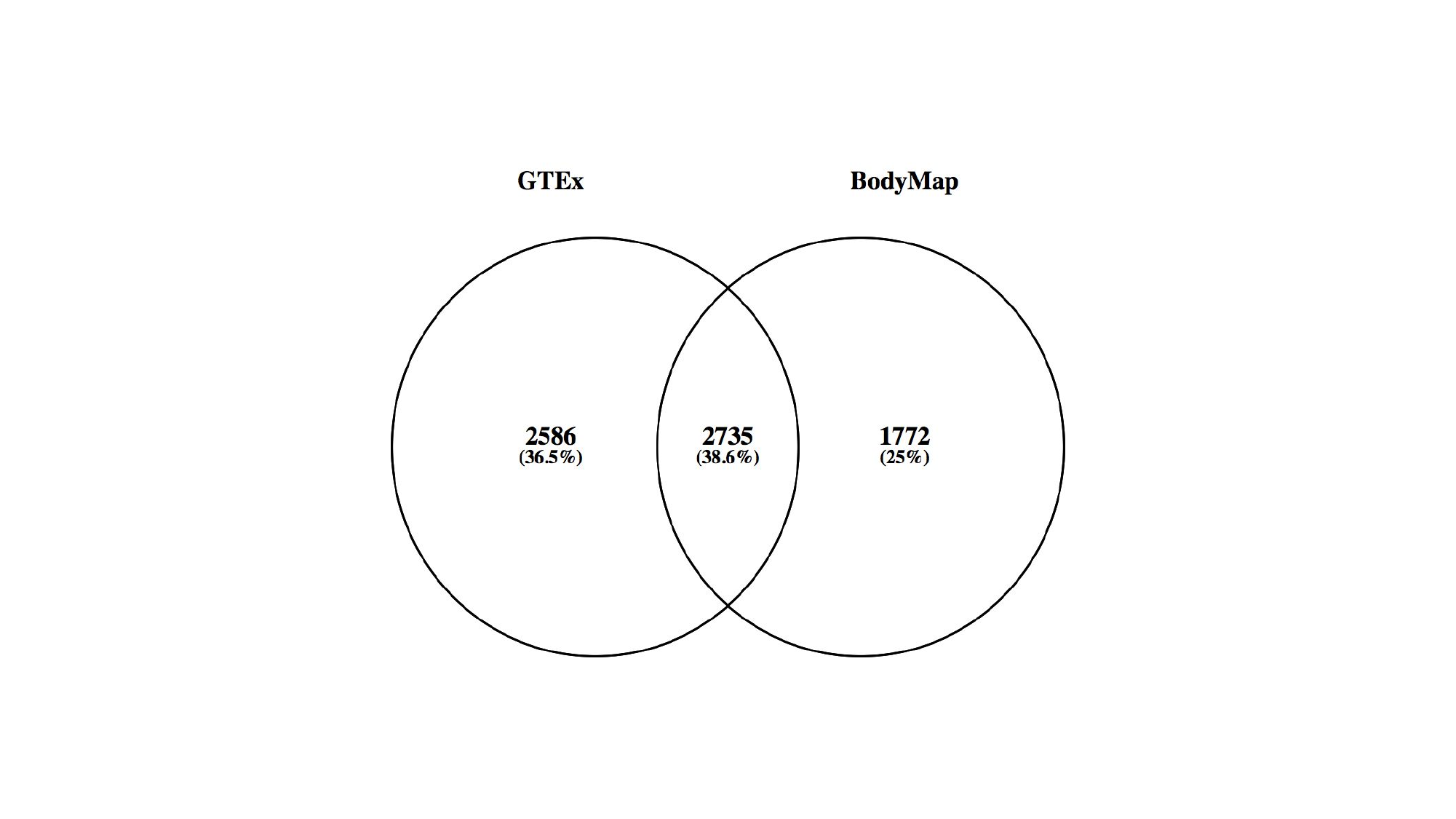

Supplement: Supplementary Figure S18 — The SEGs identified by the identical method but different datasets exhibited significant discrepancy. Even using the same method, the specifically expressed genes identified by different datasets still showed considerable levels of discrepancy. These two gene sets were downloaded from a recent SEGs study [3]. [file mmc19.pptx]

## Slide 1
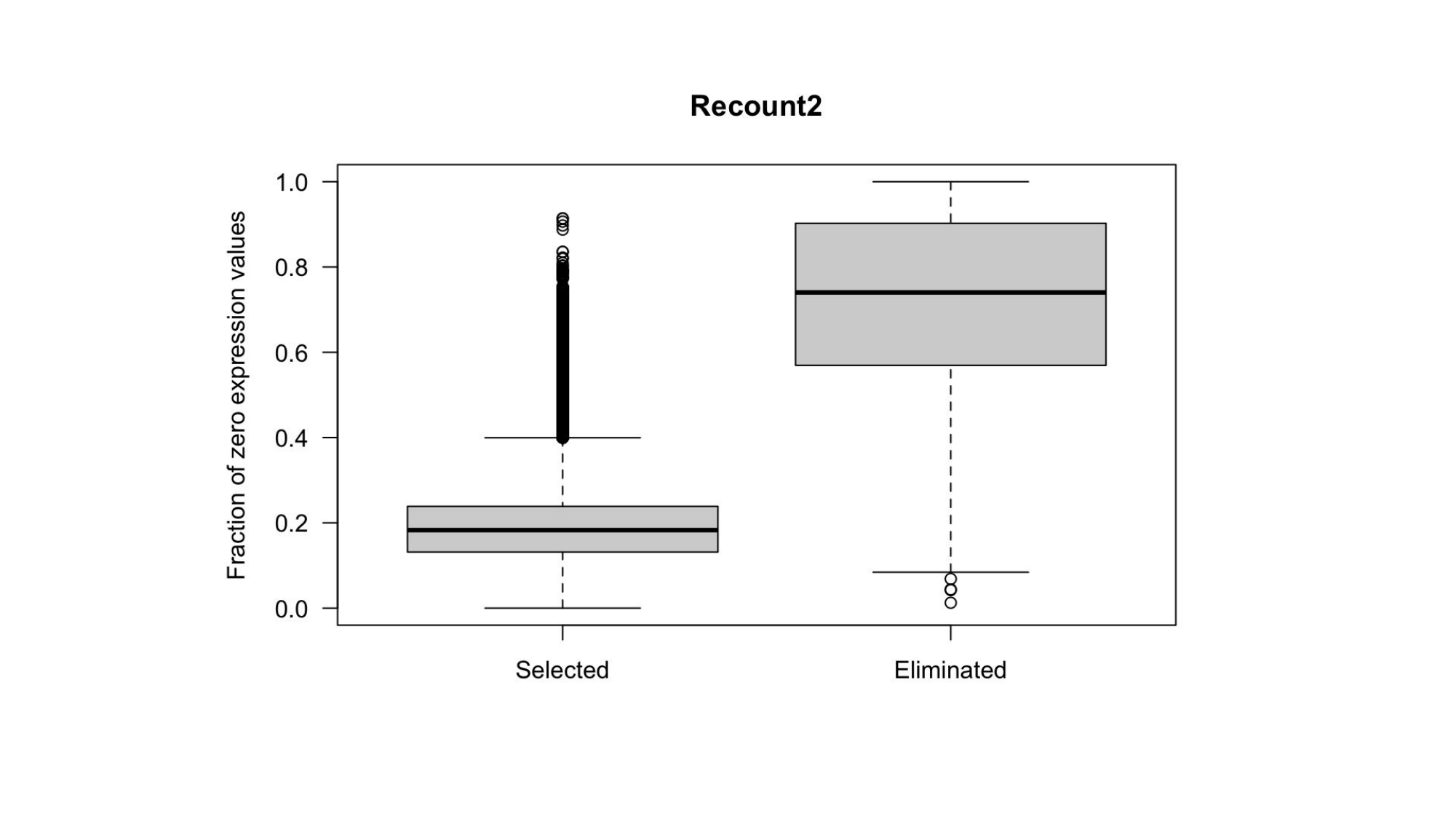

Supplement: Supplementary Figure S19 — Transcriptome profile quality control for Recount2 dataset. A transcriptome was considered as a low-quality profile if any of 3 low-expression internal reference genes (GUSB, HPRT1, and HMBS) had expression measurements of zero and was eliminated for further analyses. We used a boxplot to compare the sparsity level between the low-quality profiles and the informative profiles. [file mmc20.pptx]

## Slide 1
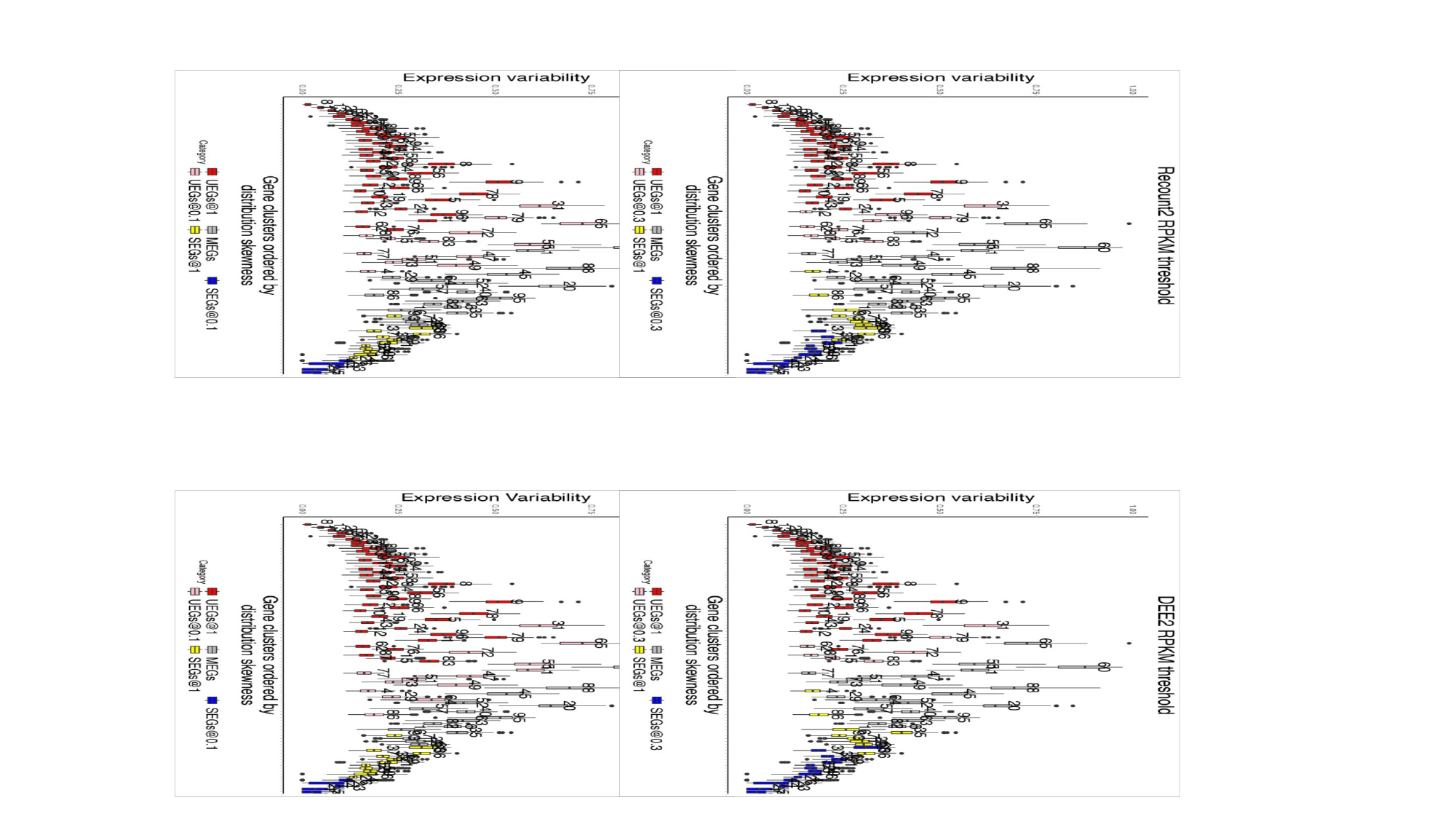

Supplement: Supplementary Figure S20 — Comparison of the global expression specificity categories identified by different normalization methods and datasets. We compared the global expression categories between different expression detection thresholds, including TPM 0.1, TPM 1.0, RPKM 0.3, and RPKM 1.0. The global expression patterns and gene clusters were obtained from recount2 quantile normalized TPM data. [file mmc21.pptx]

## Slide 1
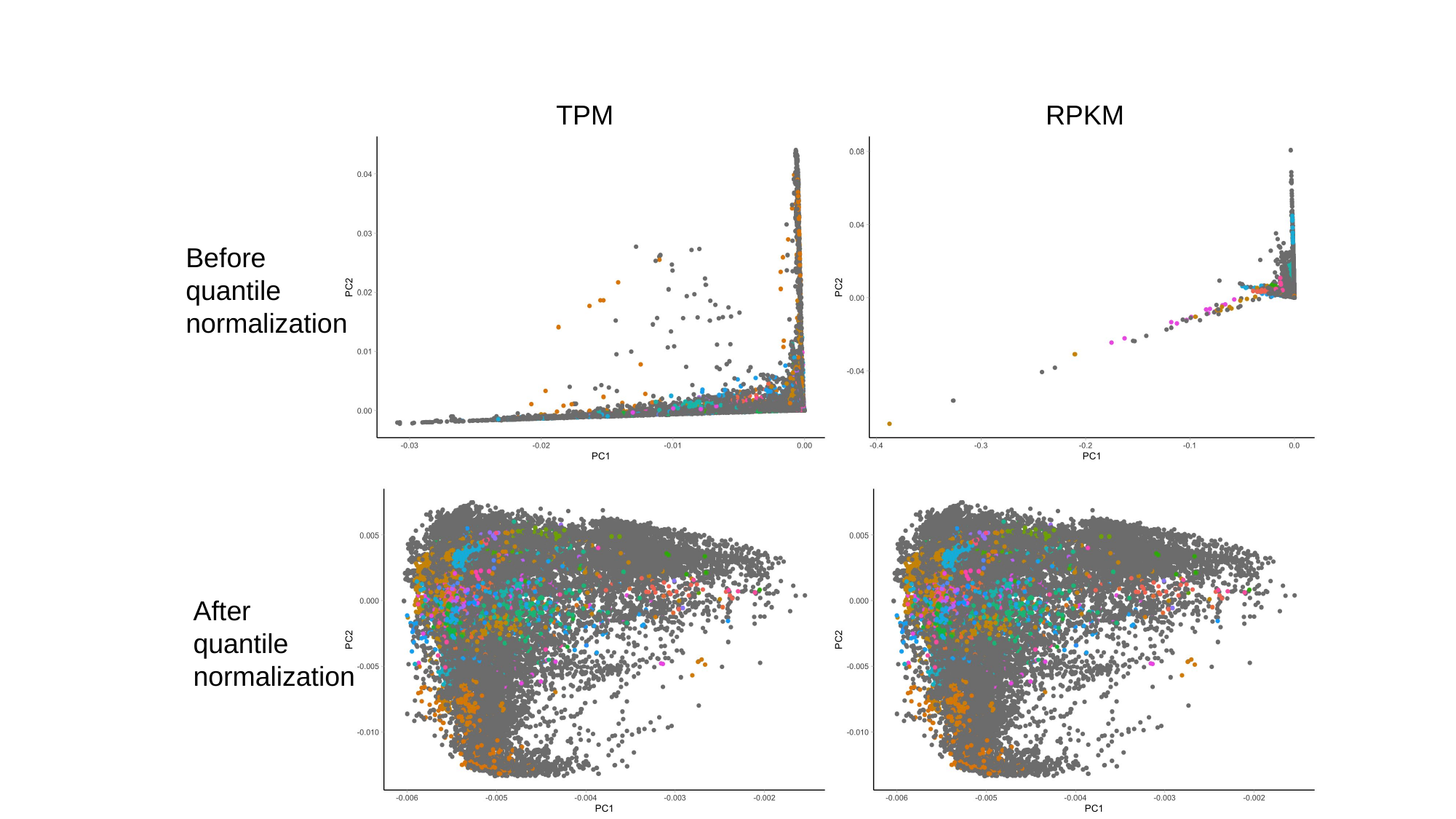

TPM
RPKM
Before
quantile
normalization
After
quantile
normalization

Supplement: Supplementary Figure S21 — Evaluating the batch effects of transcriptome profiles, onlinePCA comparison. After quantile normalization, we observed that the data points (recount2 samples) reasonably repopulate the entire transcriptome space. It implies the quantile normalized data significantly reduced the batch effects. [file mmc22.pptx]

## Slide 1
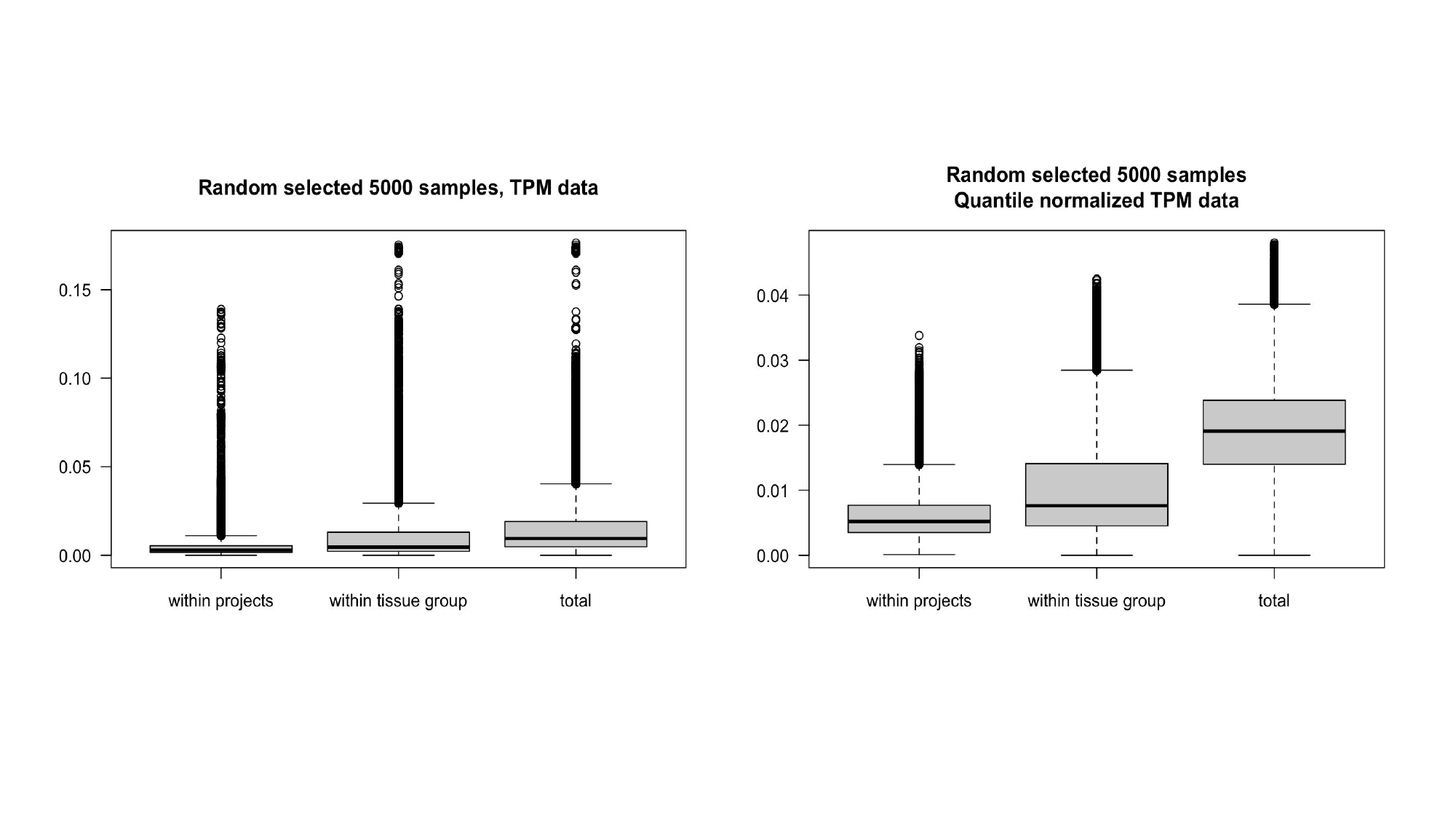

Supplement: Supplementary Figure S22 — Evaluating the batch effects of transcriptome profiles, compare within-group similarity. We randomly selected 5000 profiles and calculated the euclidean distance between data points that within-tissue-group, within-projects, and total background. We observed that the profiles from the same projects show relatively higher similarity, but the quantile normalized data significantly reduced the number of outliers. It implies that the quantile normalization method can remove most, but not necessarily all, of the variance attributed to batch. [file mmc23.pptx]

## Slide 1
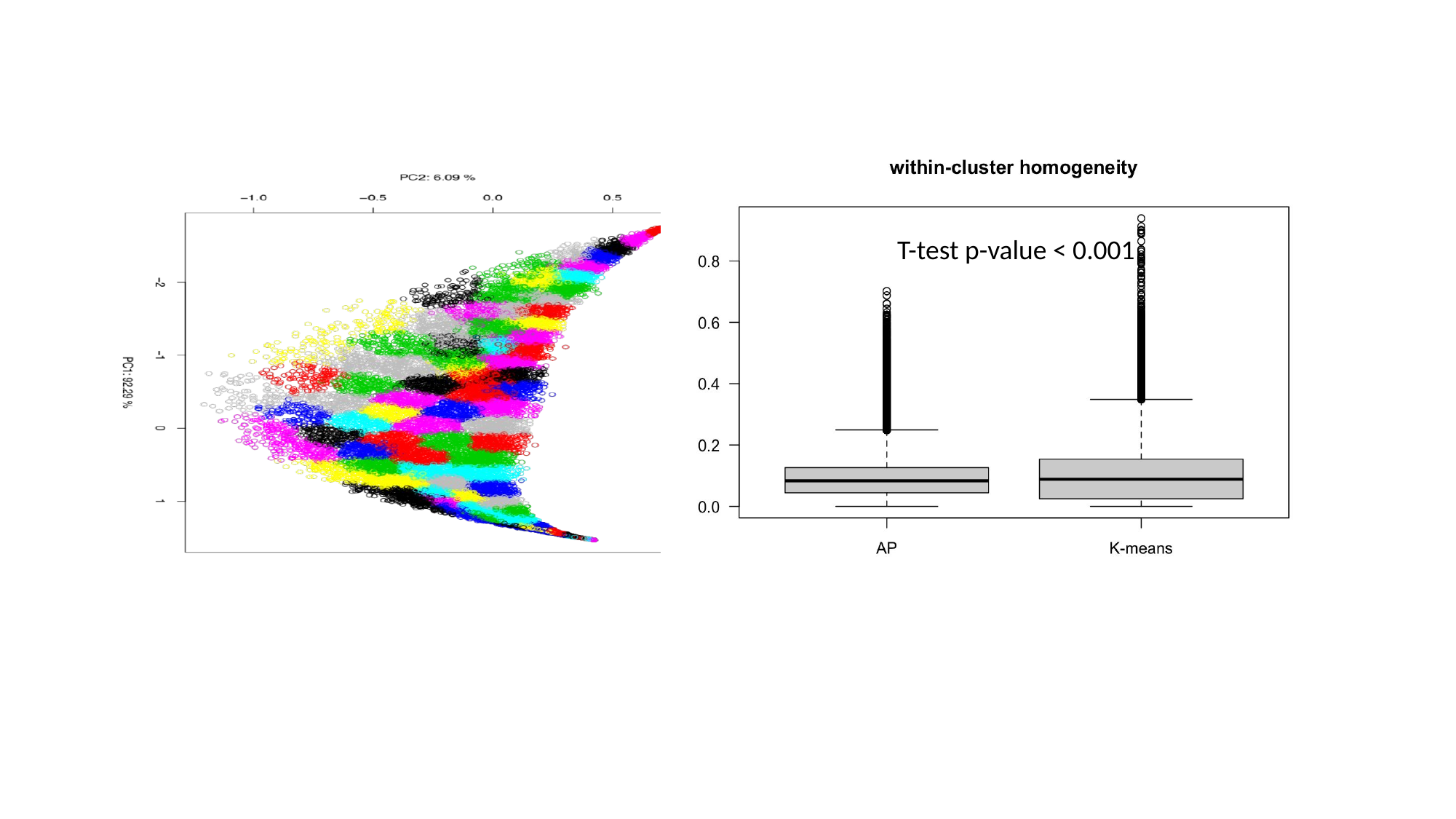

T-test p-value < 0.001

Supplement: Supplementary Figure S23 — Comparison of within-cluster homogeneity between affinity propagation and k-mean method. The left figure is the clustering results of the k-means method with the sample cluster number (96) of the AP method. The right figure shows that the AP method yielded better within-cluster homogeneity than the K-means method. AP, affinity propagation. [file mmc24.pptx]

## Slide 1
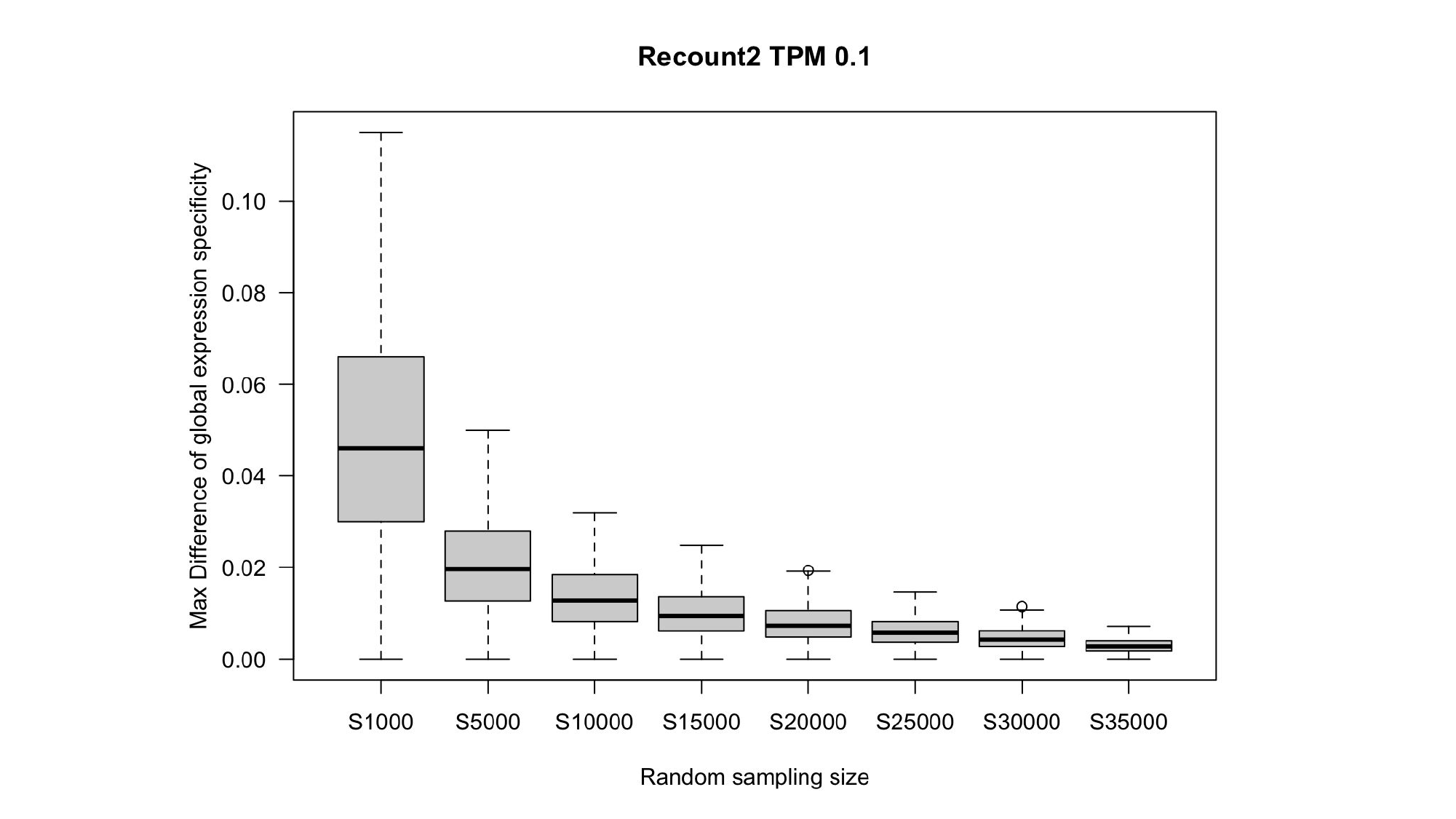

Supplement: Supplementary Figure S24 — Estimate the impacts of sample size on global expression specificity. We conducted 100 times random sampling for each gene and a series of sampling sizes (x-axis), including 1000, 5000, 10000, 15000, 20000, 25000, 25000, 30000, and 35000. The global expression specificity becomes stable as the sampling size approaches 35,000. The median maximal difference of global expression specificity is 0.0027 (0.25% of total range). [file mmc25.pptx]

## Slide 1
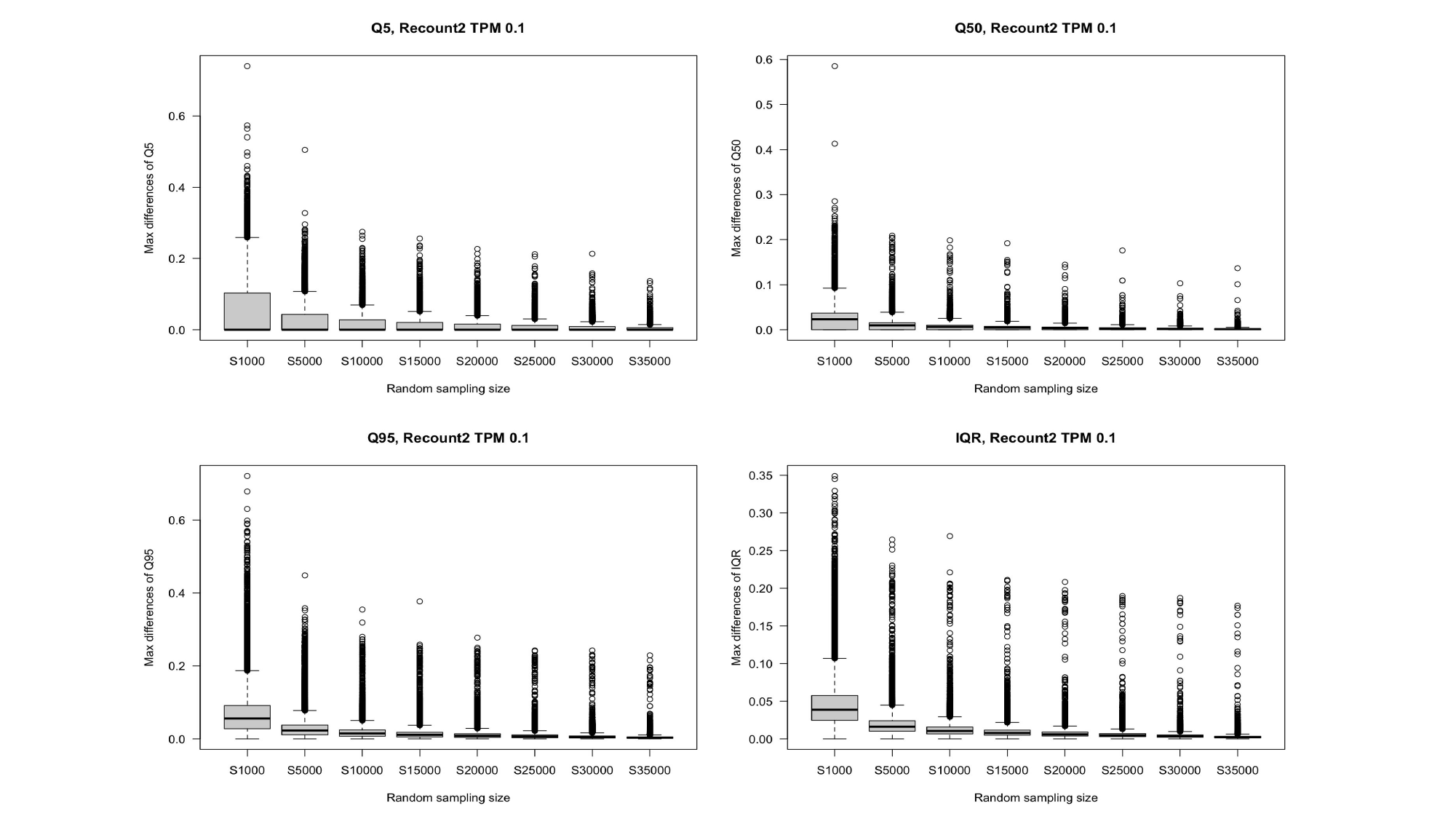

Supplement: Supplementary Figure S25 — Estimate the impacts of sample size on global distribution attributes. We conducted 100 times random sampling for each gene and sampling size (x-axis), including 1000, 5000, 10000, 15000, 20000, 25000, 25000, 30000, and 35000. The 4 major distribution attributes become stable as the sampling size approaches 35,000. The median maximal difference of these attributes is less than 0.004 (0.4% of total range). [file mmc26.pptx]

## Slide 1
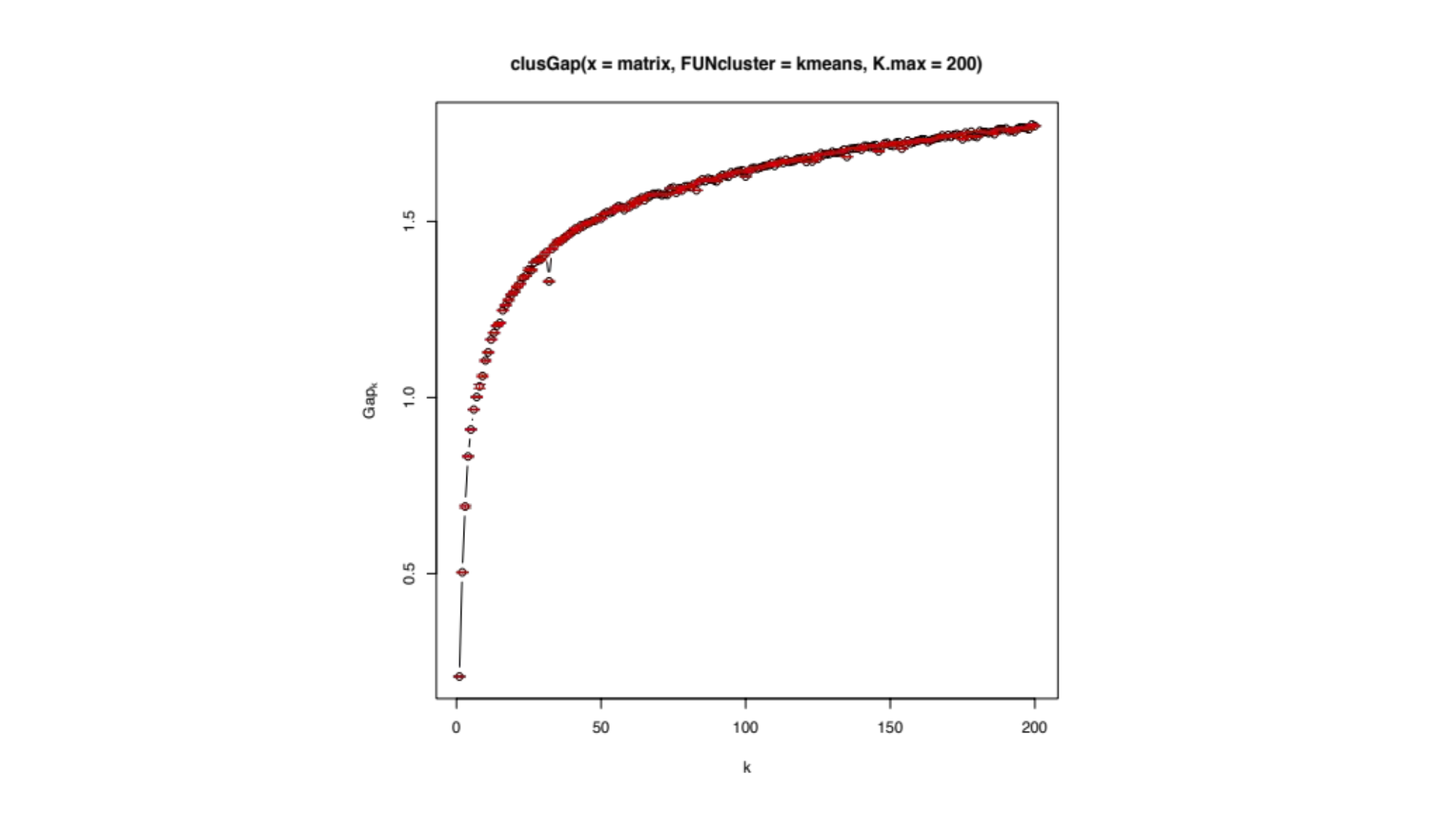

Supplement: Supplementary Figure S26 — Evaluation of the number of clusters by gap statistics. The quantile normalized TPM data with K-means clustering method was used to calculate this gap statistics. Generally, cluster number with maximum gap statistic value corresponds to the optimal number of clusters. As k increases, the gap statistics show continuous and smooth growth. However, we did not observe a clear elbow point. [file mmc27.pptx]
