# Supplementary material for: Comprehensive Analysis of Ubiquitously Expressed Genes in Humans from A Data-driven Perspective
Source: Genomics Proteomics Bioinformatics. 2022 May 13;21(1):164–76. doi: 10.1016/j.gpb.2021.08.017 (PMC10373092; doi:10.1016/j.gpb.2021.08.017)
Supplement: Supplementary Table S1 [file mmc28.docx]

### **Table S1 Phenotypic composition of analyzed recount2 transcriptome profiles**

| **UBERON term*** | **Sample size** |
| --- | --- |
| Others | 26,202 (65.73%) |
| Musculoskeletal system | 3945 (9.90%) |
| Hemolymphoid system | 3465 (8.69%) |
| Nervous system | 2978 (7.47%) |
| Digestive system | 1070 (2.68%) |
| Reproductive system | 666 (1.67%) |
| Immune system | 338 (0.84%) |
| Sensory system | 175 (0.44%) |
| Renal system | 161 (0.40%) |
| Endocrine system | 115 (0.29%) |

*Note*: *, sematic terms were annotated by MetaSRA database.
