# Supplementary material for: Comprehensive Analysis of Ubiquitously Expressed Genes in Humans from A Data-driven Perspective
Source: Genomics Proteomics Bioinformatics. 2022 May 13;21(1):164–76. doi: 10.1016/j.gpb.2021.08.017 (PMC10373092; doi:10.1016/j.gpb.2021.08.017)
Supplement: Supplementary Table S2 [file mmc29.docx]

### **Table S2 Sample types of analyzed recount2 transcriptome profiles**

| Sample type* | Sample size |
| --- | --- |
| Tissue | 16,872 (42.32%) |
| Cell line | 13,949 (34.99%) |
| Primary cells | 3,532 (8.86%) |
| In vitro differentiated cells | 3,045 (7.64%) |
| Stem cells | 1,974 (4.95%) |
| Induced pluripotent stem cells | 233 (0.58%) |
| Unknown | 93 (0.23%) |

*Note*: *, sample type was predicted by MetaSRA database.
