# Supplementary material for: Comprehensive Analysis of Ubiquitously Expressed Genes in Humans from A Data-driven Perspective
Source: Genomics Proteomics Bioinformatics. 2022 May 13;21(1):164–76. doi: 10.1016/j.gpb.2021.08.017 (PMC10373092; doi:10.1016/j.gpb.2021.08.017)
Supplement: Supplementary Table S7 [file mmc34.docx]

### **Table S7 The number of genes by each variability interval**

|  | Expression variability | | | | | |
| --- | --- | --- | --- | --- | --- | --- |
|  | **0−0.2** | **0.2−0.4** | **0.4−0.6** | **0.6−0.8** | **0.8−1.0** | **Total** |
| Total genes | 15,379 (61.61%) | 7593 (30.42%) | 1628 (6.52%) | 316 (1.27%) | 47 (0.19%) | 24,963 |
| Skewness <= 0 | 6331 (60.27%) | 3,468 (33.01%) | 590 (5.62%) | 105 (1%) | 11 (0.1%) | 10,505 (42.08%) |
| Q10 >= 0.1 | 6240 (64.38%) | 3160 (32.6%) | 284 (2.93%) | 8 (0.08%) | 0 (0%) | 9692 (38.83%) |
| Q20 >= 0.1 | 6835 (56.93%) | 4314 (35.94%) | 764 (6.36%) | 91 (0.76%) | 1 (0.01%) | 12,005 (48.09%) |
| 2011 UEGs ARRAY [10] | 1773 (86.7%) | 257 (12.57%) | 11 (0.54%) | 3 (0.15%) | 1 (0.05%) | 2045 (8.19%) |
| 2009 UEGs SEQ [8] | 5175 (66.43%) | 2325 (29.85%) | 254 (3.26%) | 32 (0.41%) | 4 (0.05%) | 7790 (31.21%) |
| 2014 UEGs SEQ [7] | 5520 (61.95%) | 2826 (31.72%) | 426 (4.78%) | 115 (1.29%) | 23 (0.26%) | 8910 (35.69%) |
| 2013 HK SEQ [5] | 3212 (84.68%) | 573 (15.11%) | 7 (0.18%) | 1 (0.03%) | 0 (0%) | 3793 (15.19%) |
| BodyMap SEGs [3] | 2160 (60.97%) | 993 (28.03%) | 268 (7.56%) | 95 (2.68%) | 27 (0.76%) | 3543 (14.19%) |
| GTEx SEGs [3] | 2364 (58.6%) | 1172 (29.05%) | 349 (8.65%) | 124 (3.07%) | 25 (0.62%) | 4034 (16.16%) |
| Essential genes [27] | 4299 (61.8%) | 2065 (29.69%) | 479 (6.89%) | 97 (1.39%) | 16 (0.23%) | 6956 (27.87%) |
| Traits genes [28] | 1593 (51.3%) | 1188 (38.26%) | 264 (8.5%) | 48 (1.55%) | 12 (0.39%) | 3105 (12.44%) |
| Genetic disease genes [29] | 8556 (53.85%) | 5579 (35.11%) | 1413 (8.89%) | 294 (1.85%) | 46 (0.29%) | 15,888 (63.65%) |
| Drugable genes [34] | 2007 (46.47%) | 1639 (37.95%) | 504 (11.67%) | 136 (3.15%) | 33 (0.76%) | 4319 (17.30%) |
| UEGs@1 category | 6187 (63.87%) | 3248 (33.53%) | 246 (2.54%) | 6 (0.06%) | 0 (0%) | 9687 (38.81%) |
| UEGs@0.1 category | 662 (25.99%) | 1127 (44.25%) | 637 (25.01%) | 113 (4.44%) | 8 (0.31%) | 2547 (10.20%) |
| MEGs category | 239 (7.59%) | 1931 (61.3%) | 744 (23.62%) | 197 (6.25%) | 39 (1.24%) | 3150 (12.62%) |
| SEGs@1 category | 1783 (58.31%) | 1274 (41.66%) | 1 (0.03%) | 0 (0%) | 0 (0%) | 3058 (12.25%) |
| SEGs@0.1 category | 6508 (99.8%) | 13 (0.2%) | 0 (0%) | 0 (0%) | 0 (0%) | 6521 (26.12%) |

*Note*: SEQ means RNAseq based study; ARRAY means microarray based study; HK is a housekeeping genes study which takes into account the variability of gene expression.
