# Supplementary material for: Comprehensive Analysis of Ubiquitously Expressed Genes in Humans from A Data-driven Perspective
Source: Genomics Proteomics Bioinformatics. 2022 May 13;21(1):164–76. doi: 10.1016/j.gpb.2021.08.017 (PMC10373092; doi:10.1016/j.gpb.2021.08.017)
Supplement: Supplementary Table S12 [file mmc39.docx]

### **Table S12 Sample types of the overrepresentation samples**

| Sample type* | Overrepresentation samples | Total samples |
| --- | --- | --- |
| Tissue | 7293 (41.71%) | 16,872 (40.43%) |
| Cell line | 7967 (45.56%) | 13,949 (34.99%) |
| Primary cells | 563 (3.22%) | 3532 (8.86%) |
| *In vitro* differentiated cells | 962 (5.50%) | 3045 (7.64%) |
| Stem cells | 579 (3.31%) | 1974 (4.95%) |
| Induced pluripotent stem cells | 53 (0.30%) | 233 (0.58%) |

*Note*: *, sematic terms were annotated by MetaSRA database.
