# Supplementary material for: Comprehensive Analysis of Ubiquitously Expressed Genes in Humans from A Data-driven Perspective
Source: Genomics Proteomics Bioinformatics. 2022 May 13;21(1):164–76. doi: 10.1016/j.gpb.2021.08.017 (PMC10373092; doi:10.1016/j.gpb.2021.08.017)
Supplement: Supplementary Table S13 [file mmc40.docx]

### **Table S13 Phenotypic composition of overrepresentation samples**

| Tissue type* | Overrepresented samples | Total samples |
| --- | --- | --- |
| Others | 12,330 (70.52%) | 26,202 (65.73%) |
| Musculoskeletal system | 1865 (10.67%) | 3945 (9.90%) |
| Hemolymphoid system | 490 (2.80%) | 3465 (8.69%) |
| Nervous system | 1339 (7.66%) | 2978 (7.47%) |
| Digestive system | 311 (1.78%) | 1070 (2.68%) |
| Reproductive system | 453 (2.59%) | 666 (1.67%) |
| Immune system | 79 (0.45%) | 338 (0.84%) |
| Sensory system | 115 (0.66%) | 175 (0.44%) |
| Renal system | 47 (0.27%) | 161 (0.40%) |
| Endocrine system | 42 (0.24%) | 115 (0.29%) |

*Note*: *, sematic terms were annotated by MetaSRA database.
